# Genome-Wide Double-Stranded RNA Sequencing Reveals the Functional Significance of Base-Paired RNAs in *Arabidopsis*

**DOI:** 10.1371/journal.pgen.1001141

**Published:** 2010-09-30

**Authors:** Qi Zheng, Paul Ryvkin, Fan Li, Isabelle Dragomir, Otto Valladares, Jamie Yang, Kajia Cao, Li-San Wang, Brian D. Gregory

**Affiliations:** 1 Department of Biology, University of Pennsylvania, Philadelphia, Pennsylvania, United States of America; 2 PENN Genome Frontiers Institute, University of Pennsylvania, Philadelphia, Pennsylvania, United States of America; 3 Genomics and Computational Biology Graduate Program, University of Pennsylvania, Philadelphia, Pennsylvania, United States of America; 4 Department of Pathology and Laboratory Medicine, University of Pennsylvania, Philadelphia, Pennsylvania, United States of America; 5 Institute on Aging, University of Pennsylvania, Philadelphia, Pennsylvania, United States of America; 6 PENN Center for Bioinformatics, University of Pennsylvania, Philadelphia, Pennsylvania, United States of America; The University of North Carolina at Chapel Hill, United States of America

## Abstract

The functional structure of all biologically active molecules is dependent on intra- and inter-molecular interactions. This is especially evident for RNA molecules whose functionality, maturation, and regulation require formation of correct secondary structure through encoded base-pairing interactions. Unfortunately, intra- and inter-molecular base-pairing information is lacking for most RNAs. Here, we marry classical nuclease-based structure mapping techniques with high-throughput sequencing technology to interrogate all base-paired RNA in *Arabidopsis thaliana* and identify ∼200 new small (sm)RNA–producing substrates of RNA–DEPENDENT RNA POLYMERASE6. Our comprehensive analysis of paired RNAs reveals conserved functionality within introns and both 5′ and 3′ untranslated regions (UTRs) of mRNAs, as well as a novel population of functional RNAs, many of which are the precursors of smRNAs. Finally, we identify intra-molecular base-pairing interactions to produce a genome-wide collection of RNA secondary structure models. Although our methodology reveals the pairing status of RNA molecules in the absence of cellular proteins, previous studies have demonstrated that structural information obtained for RNAs in solution accurately reflects their structure in ribonucleoprotein complexes. Furthermore, our identification of RNA–DEPENDENT RNA POLYMERASE6 substrates and conserved functional RNA domains within introns and both 5′ and 3′ untranslated regions (UTRs) of mRNAs using this approach strongly suggests that RNA molecules are correctly folded into their secondary structure in solution. Overall, our findings highlight the importance of base-paired RNAs in eukaryotes and present an approach that should be widely applicable for the analysis of this key structural feature of RNA.

## Author Summary

At the heart of RNA functionality, maturation, and regulation is the formation of intricate secondary structures that are dependent on specific nucleotide base-pairing interactions encoded within their sequences. These interactions can either be within (intra-molecular) or between (inter-molecular (heteroduplex)) RNA molecules. Although it is clear that secondary structure is abundantly important for the functionality and regulation of RNAs, comprehensive base-pairing interaction data are completely lacking for the majority of these molecules. To address this, we have developed a new approach for studying the base-pairing interactions of RNA molecules by marrying classical nuclease-based structure mapping techniques with high-throughput sequencing technology. We have used this approach to identify known and novel substrates of the base-paired RNA producing enzyme RNA–DEPENDENT RNA POLYMERASE6, reveal conserved functionality within introns and both 5′ and 3′ untranslated regions (UTRs) of mRNAs, uncover a novel population of functional RNAs, and produce a genome-wide collection of RNA secondary structure models by identifying the base-pairing interactions within each RNA molecule. Our findings demonstrate that our methodology should be widely applicable for the identification and analysis of base-paired RNAs in all biological organisms.

## Introduction

Recent discoveries reveal that RNAs perform a variety of tasks—ranging from the regulation of gene expression (e.g. small RNAs (smRNAs), and riboswitches) to catalytic activities (e.g. group I self-splicing introns)—and indicate that this functionality is intimately linked to their three-dimensional structure [1]–[5]. Correct secondary structure is also central to the proper regulation and maturation of RNA molecules [2], [3], [6], [7]. RNAs fold into their three-dimensional structures through specific base-pairing interactions (double-stranded RNA (dsRNA)) that are encoded within their sequence [2], [3], [6], [7]. These interactions can either be within (intra-molecular) or between (inter-molecular (heteroduplex)) RNA molecules. Although it is clear that secondary structure is abundantly important for the functionality and regulation of RNAs, comprehensive base-pairing interaction data are completely lacking for the majority of these molecules [3].

The recent discovery that RNA silencing pathways play a significant role in gene regulation has brought attention to a vast evolutionarily conserved post-transcriptional regulatory network dependent on self and foreign base-paired RNAs (dsRNAs) [8]–[10]. In RNA silencing, production of heteroduplex dsRNA or self-complementary fold-back structures gives rise to smRNAs through the activity of DICER or DICER-LIKE (DCL) RNase III-type ribonucleases [9]–[12]. In eukaryotes, smRNAs consist of microRNAs (miRNAs) and several classes of endogenous small interfering RNAs (siRNAs), which are differentiated from one another by their distinct biogenesis pathways and the classes of genomic loci from which they arise [8]. These smRNAs are the sequence-specific effectors of RNA silencing, and direct the negative regulation or control of genes, repetitive sequences, viruses, and mobile elements through inter-molecular base-pairing interactions [13], [14]. Overall, base-paired RNAs are at the core of both the biogenesis and function of all eukaryotic small silencing RNAs, emphasizing the importance of base-paired RNA in regulating gene expression.

In plants and several other organisms, there are numerous classes of endogenous and exogenous siRNAs that are processed from long dsRNA molecules synthesized by an RNA-dependent RNA polymerase (RDR) [8]–[10], [15]. The first RDR to be functionally identified as an RNA silencing pathway component in *Arabidopsis thaliana*, was RDR6 [16], [17]. RDR6 was initially uncovered due to its ability to utilize aberrant RNAs produced by transgenes as substrates for dsRNA synthesis [16]–[18]. These dsRNA molecules are subsequently converted by DCL4 into siRNAs that silence the transgenes [19]–[23]. More recently, RDR6 has been demonstrated to function in the biogenesis of endogenous smRNA populations [8], [20], [24]–[26]. One example is trans-acting siRNAs (tasiRNAs), which are processed from regions of non-coding RNAs known as *TRANS-ACTING siRNA* (*TAS*) transcripts [20], [25]–[27]. Biogenesis of tasiRNAs is initiated by siRNA or miRNA-mediated cleavage of the *TAS* transcript [20], [25]–[27]. The cleaved *TAS* transcript is then converted by RDR6 to dsRNA [20], [25]–[27], which is subsequently cleaved by DCL4 into phased 21 nucleotide (nt) siRNAs [20]–[23], [28].

Here, we describe a novel, genome-wide, high-throughput sequencing-based method, which we term dsRNA-seq, that can specifically interrogate base-paired (dsRNA) RNA molecules, and use this approach to identify and characterize ∼200 novel, smRNA-producing substrates of the dsRNA-synthesizing enzyme RDR6. Additionally, we find that mRNAs encoding proteins with functions in nucleic acid-based processes have a tendency to be highly structured. Making use of a seven-way comparative genomic approach, we demonstrate that the dsRNA-seq methodology can identify functionally conserved portions of UTRs (3′ and 5′), introns, transposable elements, as well as novel, structured RNA molecules throughout the *Arabidopsis* genome. Finally, we exploit the ability of dsRNA-seq to capture intra-molecular base-pairing interactions to produce mRNA secondary structural models on a genome-wide scale.

## Results/Discussion

### A novel approach to interrogate the dsRNA component of the *Arabidopsis* transcriptome

To obtain a transcriptome-wide view of base-paired RNA (dsRNA) in unopened flower buds of *Arabidopsis thaliana* Col-0 ecotype (hereafter referred to as wild-type Col-0), we married classical nuclease-based structure mapping techniques [29], [30] with high-throughput sequencing technology (see Figure S1A, and Materials and Methods for details). We characterized the dsRNA component of the *Arabidopsis* transcriptome after one round of ribosomal RNA (rRNA)-depletion, and obtained 15,499,789 raw reads representing 4,802,974 non-redundant (NR) sequences with an average clone-abundance of 3.2 (Accession #: GSE23439). (The size distributions for this dataset can be seen in Figure S3A.)

**Figure 1:**
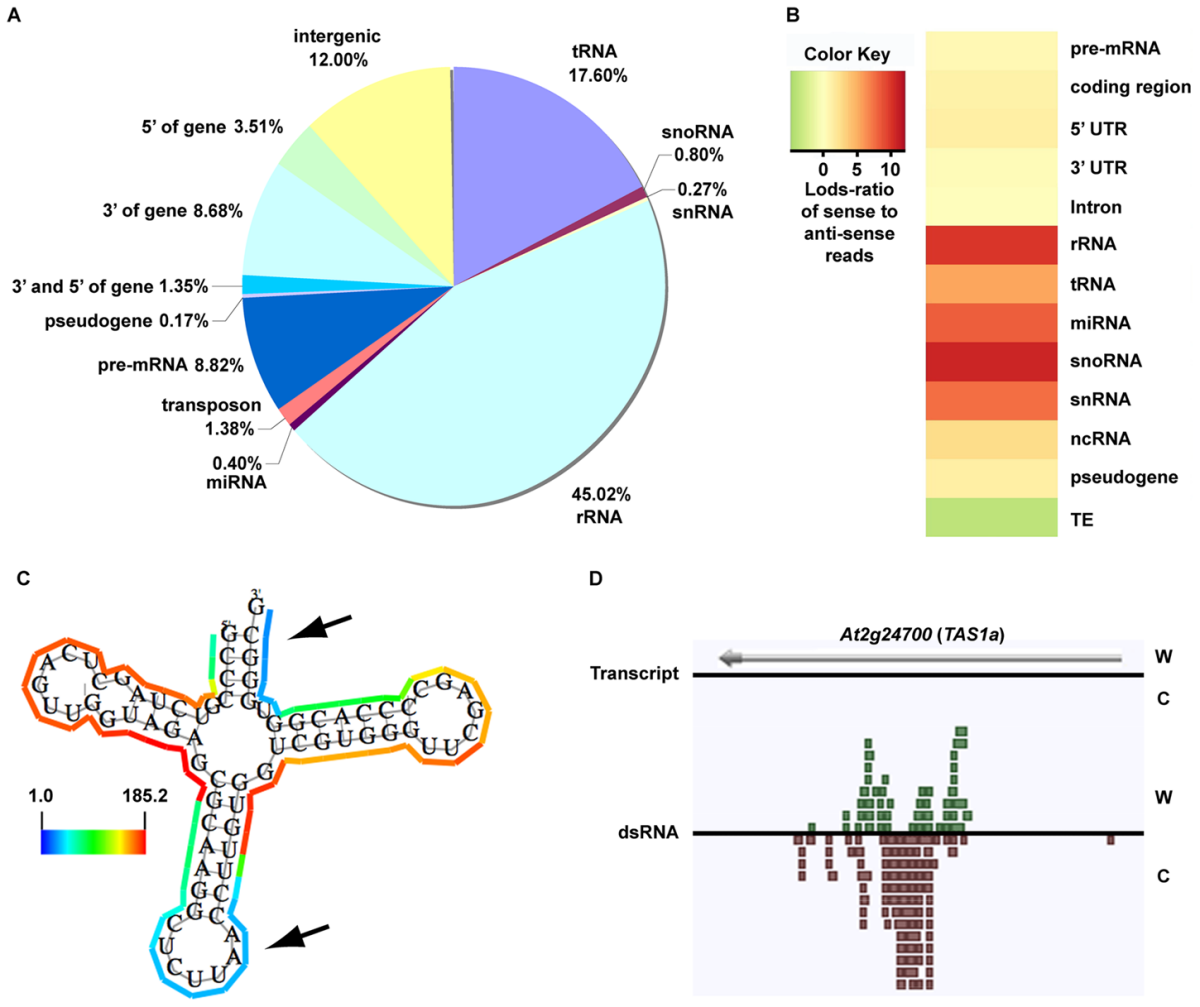
The dsRNA component of the *Arabidopsis* transcriptome. (A) Classification of genome-matching dsRNA-seq reads. (B) The heatmap indicates the strand bias of dsRNA-seq reads with respect to specific classes of RNA molecules. The color intensities indicate the degree of strand bias as specified by a log-odds ratio (Lods-ratio) value of sense/anti-sense mapping reads (red, sense; green, antisense; yellow, unbiased). TE, transposable element. (C) Model of secondary structure for an *Arabidopsis* tRNA (*At1g16100*) predicted using X-ray crystallography structure information [47]. Colored lines surrounding the model indicate the dsRNA-seq read counts that are normalized by the length of sequenced bases for each tRNA nucleotide (see scale bar for corresponding values). Black arrows specify the anti-codon loop and amino acid acceptor stem of the tRNA. (D) An intermolecular base-paired RNA molecule, *At2g24700* (*TAS1a*), identified by dsRNA-seq. Screenshot from http://tesla.pcbi.upenn.edu/annoj_at9/. W (green bars) and C (red bars) indicate sequence reads from Watson and Crick strands, respectively.

As expected, we found that the majority of our dsRNA sequencing reads corresponded to highly structured classes of RNA molecules (e.g., rRNA, tRNA, snoRNA, snRNA, etc.), smRNA-producing loci (e.g., miRNAs), and repetitive elements (e.g., transposons) (Figure 1A). We also found a large proportion of dsRNAs that correspond to protein-coding transcripts, which likely represent the self-complementary, base-pairing regions that form the secondary structure of mRNA molecules (Figure 1A). It is noteworthy that dsRNA-seq data mapped to all portions of protein-coding mRNAs, including introns, exons, and both (3′ and 5′) UTRs. Therefore, the dsRNA-seq methodology can identify base-paired regions within both mature and preprocessed mRNA molecules. (For this reason, we refer to protein-coding mRNAs within this manuscript as pre-mRNA.) Overall, our dsRNA-seq approach is robustly biased towards classes of RNA molecules that are highly base-paired in nature, which strongly suggests that this approach is interrogating the desired component of the transcriptome with a stringently estimated false discovery rate (FDR) of ≤0.067 (see Text S1).

The strand-specific nature of dsRNA-seq affords the opportunity to distinguish between intra-molecular fold-back dsRNAs (16.6% of total identified dsRNAs; example tRNA in Figure 1C) and inter-molecular heteroduplex molecules (83.4% of total identified dsRNAs; example in Figure 1D). To determine the strand bias for the different classes of RNAs captured by dsRNA-seq, we interrogated the ratio of sense versus anti-sense sequence reads. As indicated by the Log-odds (Lods) values of sense to antisense reads, the majority of RNA classes were strongly enriched for sense-strand reads, especially for the non-coding RNA classes (rRNA, tRNA, snoRNA, etc.) (Figure 1B). Specifically, functional RNAs (tRNA, miRNA, snoRNA, snRNA, and rRNA) were between 100–1000 fold enriched for the sense compared to the antisense-strand (Figure 1B). Conversely, we identified a strong anti-sense bias in our dsRNA-seq data for transposable element-derived sequences (Figure 1B). This may reflect an amplification of the antisense transposon sequence by an RDR to initiate production of siRNAs and subsequent RNA silencing of these mobile elements. For protein coding regions (exons) and 5′ UTRs of mRNAs, there was a significant sense-strand bias (∼16-fold), which was diluted for introns or 3′ UTRs of these RNA molecules. We suspect that the existence of many overlapping genes and non-coding RNAs (tRNAs, snRNAs, and snoRNAs) on the strand opposite to introns or 3′ UTRs is the confounding factor. This hypothesis is consistent with the stronger sense-strand bias in coding regions of mRNAs (Figure 1B), which have an extremely low probability of overlapping with expressed elements on the opposite strand. Additionally, there are numerous instances of 3′ end overlapping transcripts, as well as snRNA, snoRNA, and tRNA loci encoded within the introns and UTRs of protein coding mRNAs throughout the *Arabidopsis* genome. Taken together, these results suggest that by using dsRNA-seq we have identified the majority of base-paired RNA molecules (Figure S1B and S1C), which encompass a surprisingly large portion of the *Arabidopsis* genome (∼14.4% (17.3 Mb)).

As described above, dsRNA-seq captured both intra- and inter-molecular base-pairing interactions (Figure 1B–1D). In fact, we found that regions of tRNAs predicted to form intra-molecularly base-paired stems corresponded to higher levels of dsRNA-seq reads than the unpaired anti-codon loop and the amino acid acceptor stem as expected (Figure 1C). Furthermore, we observed dsRNAs that corresponded to both the Watson and Crick strands of the genome for a known substrate of the intermolecular dsRNA-synthesizing RDR6 (Figure 1D). Taken together, these results suggest that dsRNA-seq can be used to differentiate intra- from inter-molecular base-pairing interactions.

### Genome-wide identification and characterization of *Arabidopsis* RDR6 smRNA–producing substrates

An ideal test to both validate and determine the utility of dsRNA-seq is to identify all known and novel substrates of *Arabidopsis* RDR6. Accordingly, we sequenced the full complement of base-paired RNA (using dsRNA-seq) and smRNA (using smRNA-seq) molecules from unopened flower buds of wild-type Col-0 and *rdr6-11* mutant (referred to hereafter as *rdr6*) plants. For wild-type Col-0, we obtained the dsRNA-seq data described above, as well as 17,340,638 raw sequence reads representing 8,575,097 non-redundant smRNA sequences (the size distributions for this smRNA dataset can be seen in Figure S3B). Additionally, we generated a total of 18,345,980 and 18,850,891 raw sequence reads representing 9,725,315 and 9,860,471 non-redundant dsRNA and smRNA sequences for *rdr6* mutant plants, respectively (the size distributions for these *rdr6* datasets can be seen in Figure S3C and S3D, respectively).

**Figure 2:**
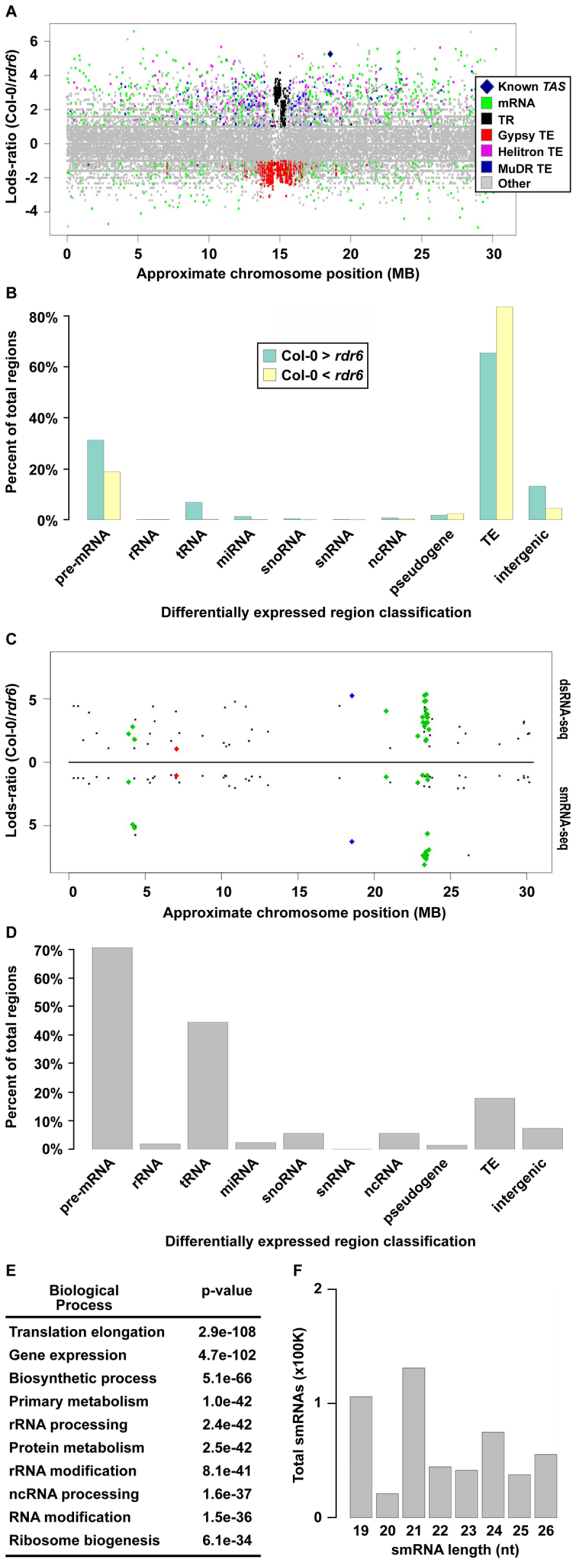
Identification of *Arabidopsis* RDR6 smRNA–producing substrates genome-wide. (A) Distribution of wild-type Col-0 compared to *rdr6* mutant 1 kb dsRNA-seq differentially expressed regions along the length of Chromosome (Chr.) 1. Each colored dot denotes a specific 1 kb region (≥2-fold and p<.001). Colored dots with positive Lods-ratio values are 1 kb regions where Col-0> *rdr6*, while negative values denote Col-0< *rdr6*. The corresponding RNA category for each colored dot can be found in the color legend box. The dark blue diamond denotes known RDR6 substrate, *TAS1b*. (B) Classification of all 1 kb regions where Col-0> *rdr6* (green bars) and Col-0< *rdr6* (yellow bars). (C) Distribution of 1 kb regions along Chr. 1 where Col-0> *rdr6* in both dsRNA- and smRNA-seq datasets (≥2-fold and p<.001). Values above black line denote Lods-ratio for dsRNA-seq regions, and values below black line denote results for smRNAs. Blue and green diamonds highlight known RDR6 substrates, while the red diamond denotes the newly identified *At1g20370*. (D) Classification of all smRNA-producing substrates of *Arabidopsis* RDR6 identified using the combination of dsRNA- and smRNA-seq. (E) The 10 most significantly enriched biological processes (and corresponding p-values) for protein-coding mRNAs that are RDR6 smRNA-producing substrates. (F) The total number of smRNAs corresponding to each indicated size class (19–26) produced from the 218 identified RDR6 substrates.

To identify potential RDR6 substrates, we used a sliding-window analysis to select 1 kilobase (kb) regions of the genome that produced ≥2-fold more dsRNA in wild-type Col-0 than in *rdr6* mutant plants with a p-value <0.001 (see Text S1). Using this approach, we identified 7,144 regions where dsRNAs are significantly depleted in *rdr6* mutant compared to wild-type Col-0 plants (Figure 2A, positive Lods-ratio values). Within these molecules, we identified 7 of 8 previously characterized *TAS* transcripts (Figure 2A, Figure S2A and S2B, blue diamonds), while the eighth was represented by a single read in both (Col-0 and *rdr6*) dsRNA-seq libraries. Additionally, we found that the majority of RDR6-dependent dsRNAs are transposable elements (mostly MuDRs and Helitrons), mRNAs, intergenic RNAs (mostly centromeric tandem repeats), or tRNAs (Figure 2A and 2B (green bars), and Figure S2B). Taken together, these results suggest that RDR6 utilizes specific classes of repetitive elements, numerous categories of functional RNAs (e.g. tRNAs, snRNAs, snoRNAs, etc.), mRNAs, and intergenic transcripts as templates for dsRNA synthesis.

Our sliding window approach also identified 7,584 dsRNAs that are significantly stabilized in *rdr6* mutant compared to wild-type Col-0 plants (Figure 2A, negative Lods-ratio values). The vast majority (>80%) of the molecules stabilized in *rdr6* mutant plants are TEs (Figure 2B, yellow bars), most of which (∼95%) are pericentrometric Gypsy-like transposons (Figure 2A and 2B (yellow bars), and S2B). We also found a number of these dsRNAs correspond to mRNAs (∼15%) and intergenic transcripts (∼4%) (Figure 2B, yellow bars). Overall, the identification of dsRNA molecules that are stabilized in *rdr6* mutant plants suggests a potential model where RDR6 antagonizes the action of other RDRs at some targets, especially at Gypsy-like transposons.

**Figure 3:**
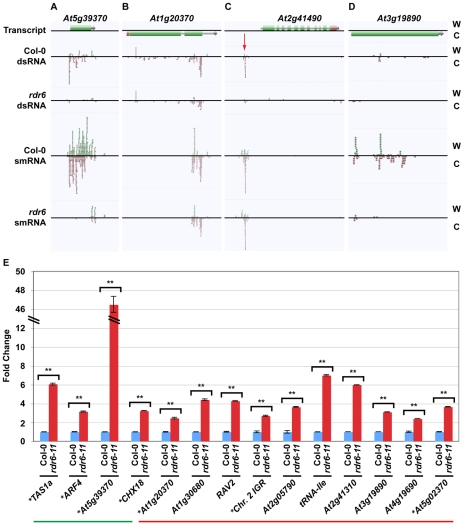
Novel smRNA–producing substrates of RDR6. (A–D) Four examples of RDR6 smRNA-generating substrates identified using the combination of dsRNA- and smRNA-seq (screenshots from http://tesla.pcbi.upenn.edu/annoj_at9/). W (green bars) and C (red bars) indicate sequence reads from Watson and Crick strands, respectively. (A) *At5g39370* (previously identified), (B) *At1g20370* (novel), (C) the intergenic region just upstream of *At2g41490* (novel), and (D) *At3g19890* (novel). (E) Random-primed RT-qPCR analysis of four previously identified and 10 novel RDR6 substrates for wild-type Col-0 and *rdr6-11* mutant plants. Error bars, ±SD. ** indicates p-value <.001. Green and red lines underline previously identified and novel RDR6 substrates, respectively. * denotes RDR6 substrates that produce phased siRNAs.

The consequence of dsRNA synthesis by RDR6 is often the subsequent formation of siRNAs [19]. Therefore, to identify those RDR6 dsRNA substrates that produce smRNAs, we identified regions that produce ≥2-fold more smRNAs in wild-type Col-0 than in *rdr6* mutant plants. These sources of smRNA were then compared with the regions of the genome that produce more dsRNA in wild-type Col-0 than in *rdr6* mutant plants, which identified 218 regions that met both criteria (Figure 2C and Figure 3A–3D; Table S1). These common regions include ∼50% (27 total) of the previously identified smRNA-producing RDR6 substrates, the majority of which were not known to be expressed in *Arabidopsis* unopened flower buds (Figure 2C and Figure S2C; Tables S1 and S2) [31]–[34]. The other 6,926 regions where dsRNAs, but not smRNAs, are significantly depleted in *rdr6* mutant compared to wild-type Col-0 plants consist of mostly MuDR and Helitron transposable elements. These results suggest that the double-stranded *MuDR*s and *Helitron*s produced by RDR6 may only constitute an insignificant subset of the smRNA-producing population of these transposons. Conversely, RDR6 synthesized *MuDR* and *Helitron* dsRNAs may simply not be processed into smRNAs.

Our analysis also revealed that the majority of highly confident smRNA-producing RDR6 substrates are mRNAs with a variety of biological functions (Figure 2D and 2E) and, surprisingly, tRNAs (Figure 2D). As expected, the identified RDR6 substrates tend to produce 21 nt smRNAs (Figure 2F). It is noteworthy that RDR6-targeted mRNAs mostly encode proteins that function in nucleic acid-based biological functions (e.g. translation, RNA processing, etc.) and regulation of gene expression (Figure 2E). Taken together, these results suggest that an RDR6-dependent RNA silencing pathway regulates multiple stages of gene expression through siRNA production in *Arabidopsis*.

The identification of tRNAs as RDR6 substrates is intriguing because it was recently suggested that the mammalian telomerase reverse transcriptase catalytic subunit (Tert) functions as a smRNA-producing RDR that can also use tRNAs as substrates [15]. Taken together, these results suggest that plant RDR6 and animal Tert are functional orthologs that can use tRNAs as substrates for production of dsRNA precursors of smRNAs. Therefore, studies of RDR6 may be informative for gaining insight into the function of mammalian RDRs, and vice versa.

In order to validate and expand our characterization of new smRNA-producing RDR6 substrates, we turned to a quantitative reverse transcription polymerase chain reaction (qRT-PCR) approach. For these loci, RDR6 is required to produce a dsRNA precursor of siRNAs (see Figure 3A–3D). Therefore, if RDR6 is not active (*rdr6* mutant plants), then the single-stranded transcripts may be stabilized. To test this hypothesis, we designed qRT-PCR primers to 14 (four known, 10 novel) identified smRNA-producing RDR6 substrates. We found that all fourteen tested loci, including the 10 newly identified RDR6 substrates (e.g. *At1g20370* (Figure 3B), the intergenic region just upstream of *At2g41490* (Figure 3C), and *At3g19890* (Figure 3D)), had higher transcript levels in *rdr6* mutant compared to wild-type Col-0 plants (Figure 3E). These results suggest that most, if not all of the 218 loci we identified using a combination of dsRNA-seq and smRNA-seq methodologies are true smRNA-producing RDR6 substrates; approximately 200 of these loci are novel (Tables S1 and S2).

Most previously identified endogenous RDR6 substrates produce phased 21 nt siRNAs [20]–[23], [28]. We found that 51 of the RDR6 substrates identified in this study also produce phased smRNAs (Table S2 and Figure S2D). This group includes 22 of the RDR6 substrates that have been previously reported [31]–[34], as well as the newly identified substrates, *At1g20370* (Figure 3B and 3E), the intergenic region just upstream of *At2g41490* (Figure 3C and 3E), and *At5g02370* (Figure 3E; Tables S1 and S2). However, we found that >75% of all endogenous smRNA-producing RDR6 substrates (167) do not produce siRNAs with any recognizable phasing, including the newly identified *At3g19890* (Figure 3D and 3E; Tables S1 and S2). These results suggest that there are multiple mechanisms by which transcripts become susceptible to RDR6-mediated silencing. In summary, our results suggest that the combination of dsRNA-seq and smRNA-seq is a highly sensitive method for identifying transcripts subject to RDR6-dependent silencing, and is likely to be useful for characterizing the substrates of other eukaryotic RDRs - such as mammalian Tert [15] - that have not been demonstrated to produce phased siRNAs.

### Identification of dsRNA “hotspots” in the *Arabidopsis* genome

**Figure 4:**
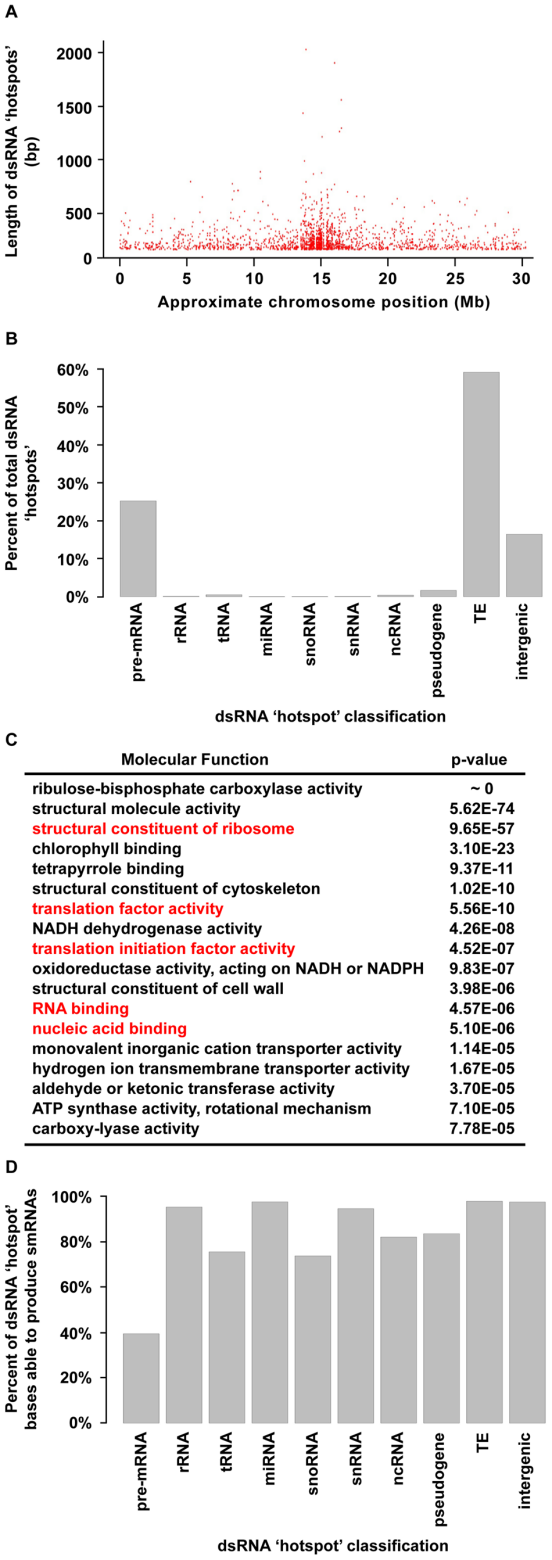
Highly base-paired segments of the *Arabidopsis* genome (dsRNA “hotspots”). (A) Approximate genomic distribution (∼100 kb resolution) and length of dsRNA “hotspots” along *Arabidopsis* Chr. 1 for wild-type Col-0. (B) Classification of dsRNA “hotspots.” TE, transposable element. (C) The 18 most significantly enriched molecular functions for protein-coding mRNAs that contain dsRNA ‘hotspots’. Red labels indicate nucleic acid biology GO categories. (D) The percent of nucleotides within dsRNA ‘hotspots’ that were found to produce smRNAs. The smRNA data used for this analysis is described in Figure S8.

We next identified regions of the *Arabidopsis* genome that are significantly enriched for base-paired RNA using the dsRNA-seq data for wild-type Col-0. For this purpose, we used a geometric distribution-based approach to identify unusually long dsRNA molecules (dsRNA ‘hotspots’) based on the average size of dsRNAs computed for each chromosome independently. This analysis revealed 9,719 dsRNA ‘hotspots’ of varying lengths scattered along the entire length of all *Arabidopsis* chromosomes (Figure 4A and Figure S4A; Tables S3 and S4). In fact, we have identified the vast majority of highly base-paired RNA molecules in the *Arabidopsis* transcriptome (Figure S9). For example, the highly repetitive, transposon-rich pericentromeric regions of the *Arabidopsis* genome were found to be a rich source of dsRNA (Figure 4A and 4B, and Figure S4A). This is not surprising because *cis* transcriptional silencing of transposons and repetitive elements in the pericentromeric regions of *Arabidopsis* chromosomes is mediated by RDR2-dependent siRNAs [35]–[38]. These findings not only substantiate that dsRNA-seq interrogates the desired portion of the transcriptome, but also suggest that, as expected, *Arabidopsis* transposons and repetitive elements are highly enriched in dsRNA on a genome-wide scale.

A classification of *Arabidopsis* dsRNA ‘hotspots’ revealed that transposons and protein-coding mRNAs are the two most highly base-paired classes of RNA molecules (Figure 4B). In fact, we identified 1949 protein-coding mRNAs that contained dsRNA ‘hotspots’ (Figure 4B), so we interrogated over-represented molecular functions for these genes using Gene Ontology (GO) analysis. Ribulose-bisphosphate carboxylase was the most significantly over-represented protein in this analysis. However, the most highly over-represented group of genes were those involved in nucleic acid biology (e.g., translation, nucleic acid binding, etc.) (Figure 4C). Interestingly, genes involved in nucleic acid metabolism are also over-represented in dsRNA ‘hotspot’-containing transcripts of *Drosophila melanogaster* and *Caenorhabditis elegans* (Q.Z. and B.D.G., unpublished data). Thus, a propensity to form complex secondary structure (self base-pairing) may be a general feature of eukaryotic transcripts that encode proteins involved in processes involving nucleic acids. This may point to a feedback regulatory mechanism that is dependent on an interaction between the proteins encoded by these transcripts and highly structured RNA intermediates.

The biogenesis of all functional small silencing RNAs (e.g. miRNAs and siRNAs) requires a dsRNA intermediate. Therefore, we determined the propensity of highly base-paired regions (dsRNA ‘hotspots’) to be processed into smRNAs (Figure 4D) using corresponding smRNA-seq data (Figure 2C; see Figure S8 for smRNA data analysis). We found that the highly base-paired regions within 9 of 10 interrogated RNA categories were extremely likely to be processed into smRNAs, the exception being pre-mRNA molecules (Figure 4D). Although these results were expected for transposable elements and miRNAs - which are known to be smRNA biogenesis substrates - it was surprising that functional RNAs (e.g. rRNA, tRNA, snRNA, etc.) also have a high likelihood of being processed into smRNAs since intramolecular base-pairing interactions are intrinsic to their function.

The evidence that highly base-paired regions of RNA molecules are frequently processed into smRNA, suggests that this process may be important for regulating the abundance of functional RNAs in *Arabidopsis* cells. Our finding that any highly base-paired molecule can be processed into smRNAs, may provide an explanation for the restriction of the miRNA biogenesis machinery to specific sites within the plant nucleus (dicing bodies) [39], [40]. An intriguing hypothesis is that the sequestration of proteins involved in miRNA biogenesis and their *MIRNA* substrates to dicing bodies provides specificity to miRNA biogenesis, while protecting other structured RNAs (e.g. rRNA) from these proteins. Our findings suggest further studies of smRNA sources in eukaryotes will reveal additional siRNA-mediated regulatory pathways, as demonstrated, for example, by the analysis of tRNA-derived RNA fragments (tRFs) in human cells [41].

### Comparative genomics of dsRNA “hotspots” reveals functionality within introns, both UTRs, and intergenic regions of the *Arabidopsis* genome

**Figure 5:**
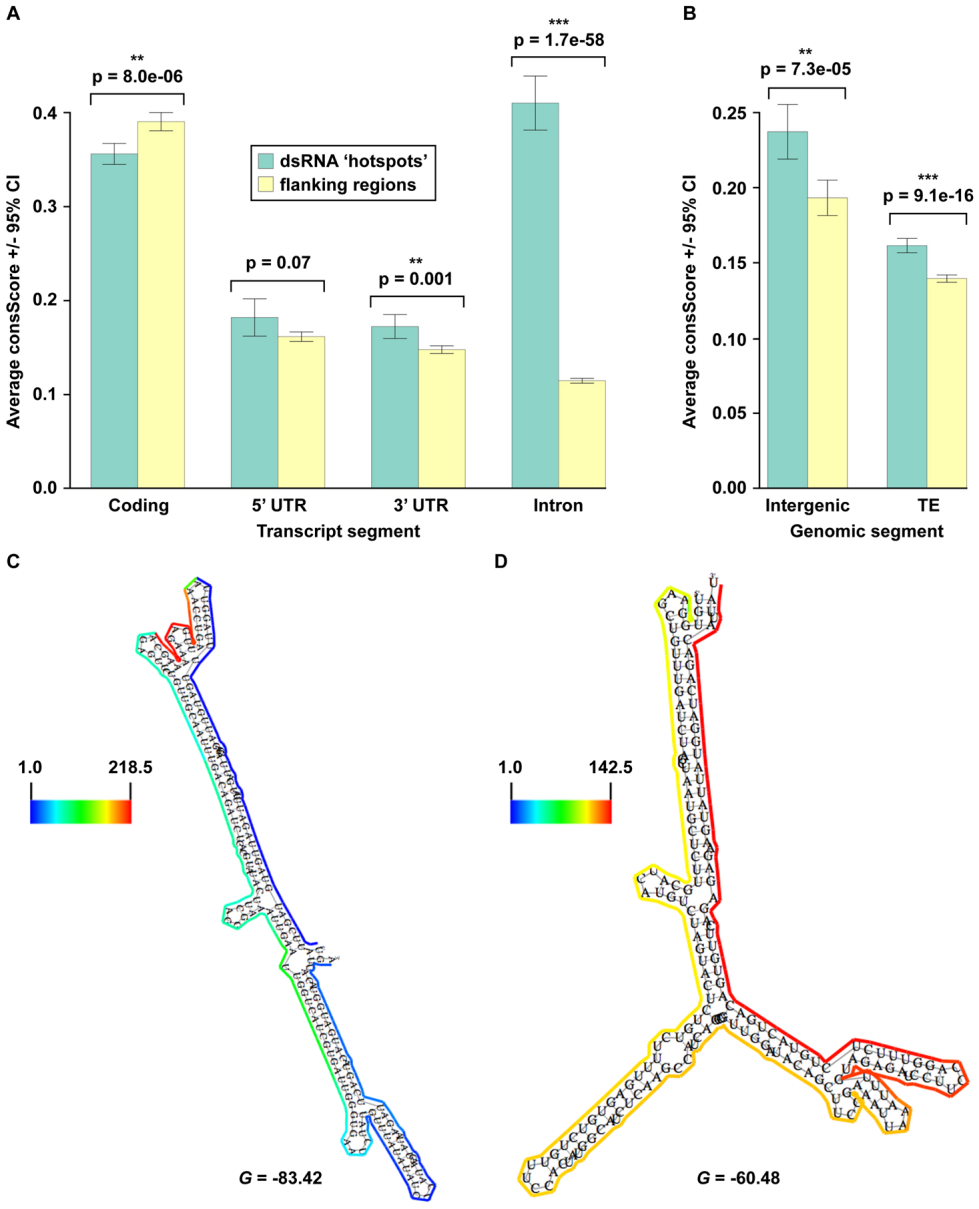
Identification of widespread, conserved functionality within non-coding portions of mRNA (introns, 3′ and 5′ UTRs), intergenic regions, and transposons. (A, B) The average conservation scores (consScore) calculated using a seven-way comparative genomics analysis of dsRNA ‘hotspots’ (green bars) or their flanking regions (yellow bars) in specific portions (coding (exons), 5′ UTR, 3′ UTR, and introns) of pre-mRNAs (A), as well as intergenic regions and transposons (TE) (B). (C, D) Models of secondary structure for *Arabidopsis* (E) *At1g67430* (nt 25262487–25262809) and (F) *At2g40650* (nt 16964129–16964413) intronic functional moieties determined by dsRNA-seq constrained parameters for RNAfold (see below) (screenshots from the structural viewer at http://tesla.pcbi.upenn.edu/annoj_at9/). The scale bar to the left of each model indicates the read counts that are normalized by the length of sequenced bases for the transcript. The multiple alignments for these conserved, intronic dsRNA ‘hotspots’ can be seen in Figure S5A and S5B. *G* denotes the Gibb's free energy value (kilocalories/mole) for the corresponding RNA secondary structure model.

Regulation and maturation of eukaryotic pre-mRNA molecules is intimately linked to the proper formation of secondary structure [2], [3], [6], [7], which suggests that base-paired regions of these molecules are likely to be functionally conserved. To test this hypothesis, we employed a seven-way comparative genomics approach that determines an average conservation score (consScore) for all bases of dsRNA ‘hotspots’ and all other sequences (‘flanking regions’) within the four structural moieties (exons, introns, and both UTRs) of every mRNA. The consScores for dsRNA ‘hotspots’ and ‘flanking regions’ were then compared to determine if base-pairing mediates evolutionary conservation of mRNAs. Using this approach, we found that dsRNA ‘hotspots’ in exons are significantly less evolutionarily conserved than ‘flanking regions’ (Figure 5A), which suggests that intra- and/or intermolecular base-pairing interactions are disfavored in the protein-coding regions of plant mRNAs.

Our comparative genomic analysis of pre-mRNA data also demonstrated that dsRNA ‘hotspots’ are significantly more conserved than ‘flanking regions’ in 3′ UTRs (p = 0.0012) and introns (p = 1.73e–58) (Figure 5A), and that highly base-paired regions within 5′ UTRs (p = .072) were more evolutionarily conserved than ‘flanking regions’, but far less significantly than in 3′ UTRs and introns. This analysis suggests the ability to base-pair is functionally important, and has been selected during plant evolution. Just as selection for protein function maintains exonic sequences, base-pairing interactions may be important for conserving functionally important moieties in non-coding regions of mRNAs. These functions may include 1) providing appropriate structure for post-transcriptional and/or translational regulation, 2) maintaining mRNA stability, 3) providing *cis*-element sites for RNA binding proteins, and/or 4) forming the processed precursors of non-coding RNAs. Similar results have been obtained for *Drosophila melanogaster* and *Caenorhabditis elegans* (Q.Z. and B.D.G., unpublished data), suggesting that the ability to base pair is a critical feature of UTRs and introns in both plants and animals. An mRNA secondary structure prediction methodology (see below) was used to obtain a folded model of two highly conserved intronic dsRNAs (see Figure S5A and S5B for alignments), and suggested that these regions are almost entirely base-paired, and fold into unique, stable secondary structures (Figure 5C and 5D). Taken together, our results reveal that dsRNA-seq identifies functionally conserved regions of 5′ and 3′ UTRs and introns transcriptome-wide, and thus provides the critical first step towards understanding how such structural moieties affect the maturation and stability of transcripts in eukaryotic organisms.

We also noticed that a number of our dsRNA ‘hotspots’ are located in transposons and portions of the genome that do not contain any known genes. Comparative analysis revealed that dsRNA ‘hotspots’ in intergenic regions (p = 7.3e–5) and transposons (p = 9.1e–16) are significantly more conserved than their flanking regions (Figure 5B). In the case of transposons, this finding was quite surprising because the majority of these repetitive elements are selectively neutral, especially for ancestral repeats (ARs) [42], [43]. However, our findings demonstrate that the highly antisense-prone transposable element dsRNA ‘hotspots’ (Figure S4C and S4D) have been undergoing a significant purifying selection compared to their ‘flanking regions’, suggesting that these portions of TEs are not selectively neutral, but have important functions in plant cells. An intriguing hypothesis is that a class of smRNAs that are integral to initiate and/or maintain the transcriptional silencing of transposable elements are processed from these conserved highly-base paired regions. Overall, these results reveal functionally conserved portions of transposons, as well as novel, structured RNAs that have not been previously identified.

### Identification and characterization of novel, highly base-paired RNAs with conserved functions in land plants

**Figure 6:**
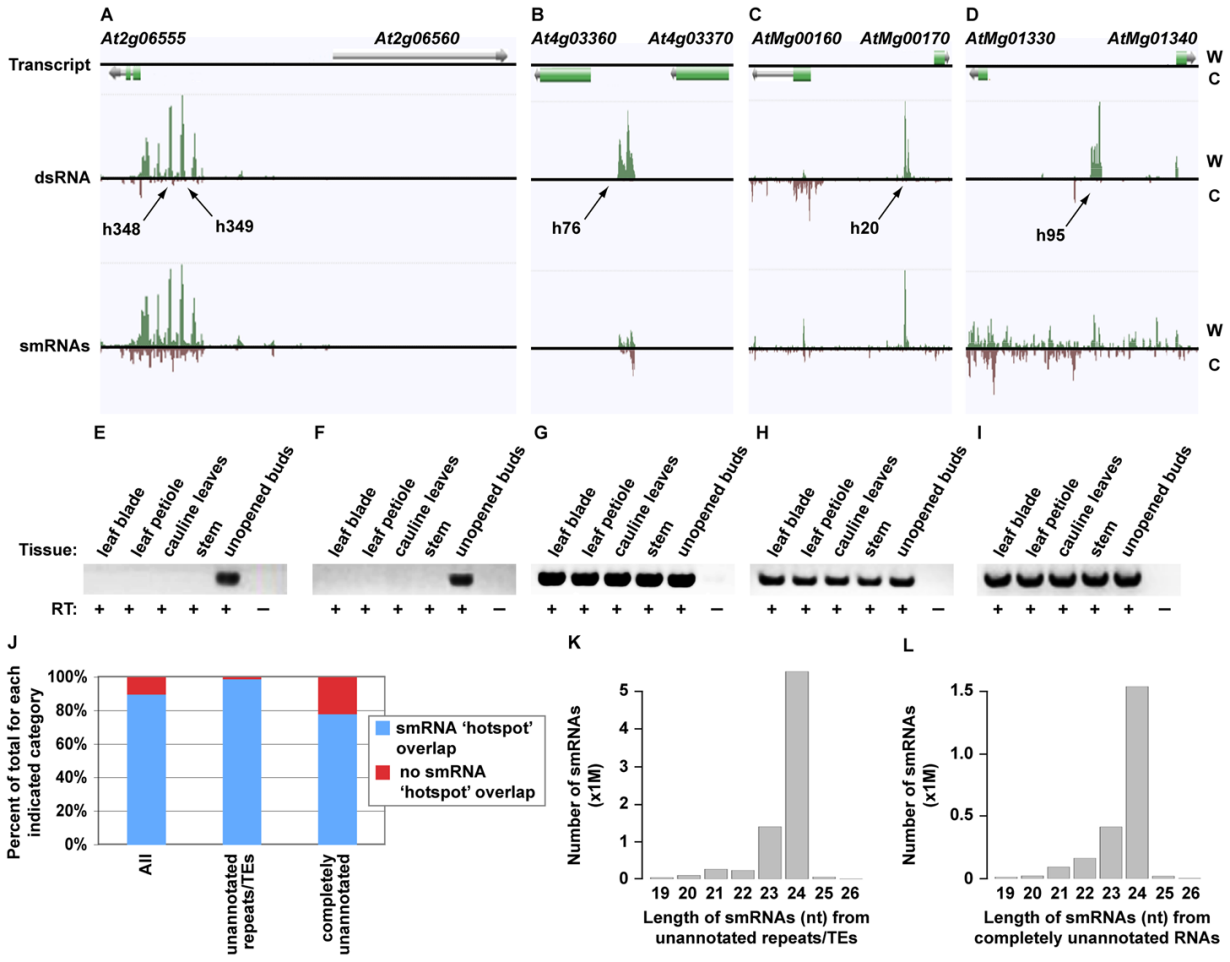
Identification of novel, highly structured RNAs using dsRNA–seq. (A–D) Four examples of intergenic, highly base-paired transcripts (screenshots from http://tesla.pcbi.upenn.edu/annoj_at9). W (red bars) and C (green bars) indicate signal from Watson and Crick strands, respectively. (A) Two intergenic dsRNA ‘hotspots’ (h348 and h349) found between *At2g06555* and *At2g06560*. (B) A novel, base-paired RNA on Chr. 4 between *At4g03360* and *At4g03370*. (C) A Chr. M intergenic dsRNA ‘hotspot’ between *AtMg00160* and *AtMg00170*. (D) An example of a new, highly structured RNA from Chr. M that lies between *AtMg01330* and *AtMg01340*. (E–I) Random-primed RT-PCR analysis of the novel, base-paired RNAs that are pictured in (A–D) using five different *Arabidopsis* tissues (leaf blade, leaf petiole, cauline leaves, stem, and unopened flower bud clusters). (E, F) correspond to h348 and h349 in (A), respectively. (G–I) correspond to (B–D), respectively. Flower bud RNA samples that were not treated with reverse transcriptase serve as controls for this experiment. (J) The percent of total new transcripts for each indicated category that do (blue bars) or do not (red bars) overlap with smRNA ‘hotspots’. There are 1,602, 897, and 705 corresponding transcription units for the All, unannotated repeats/TEs, and completely unannotated categories, respectively. TE, transposable element. (K) The number of smRNAs corresponding to each indicated size class (19–26) produced from the unannotated repeats/TEs. (L) The number smRNAs corresponding to each indicated size class (19–26) produced from the completely unannotated transcription units.

We identified a total of 1602 novel transcripts, ∼60% of which are unannotated transposable elements and/or simple repeats (Figure 6J; Tables S5 and S6). The other >700 transcripts represent newly identified RNAs. To determine the function of these 1602 transcripts we looked for the presence of these sequences in our flower bud smRNA dataset (see Figure S8 for smRNA analysis). 1437 (89.7%) of the novel RNAs overlapped regions of the genome that produce significant quantities of smRNAs (smRNA ‘hotspots’, Figure S8) (Figure 6 and Figure S6; Tables S5 and S6). Specifically, >98% of the unannotated transposable elements and/or simple repeats and ∼79% of the entirely novel RNAs produced smRNAs, respectively (Figure 6J). Most smRNAs from these transcripts were 24 nt in length (Figure 6K and 6L). In *Arabidopsis*, this size class is highly correlated with DNA methylation and heterochromatin formation [44], suggesting that these loci produce 24 nt smRNAs that direct transcriptional silencing.

To validate our sequencing data and further interrogate the newly identified transcription units, we characterized several of these RNAs by reverse transcription (RT) polymerase chain reaction (RT-PCR) in five different *Arabidopsis* tissues (leaf blade, leaf petiole, cauline leaves, stem, and unopened flower buds). We selected four loci that do (see Figure 6A and 6C; Figure S6A, S6C, and S6E; Table S5) and seven RNAs that do not (Figure 6B and 6D; Figure S6B, S6D, S6F, S6G, S6K, S6L, and S6M; Table S5) produce statistically significant amounts of smRNAs (11 total transcripts). As expected, all 11 of these RNAs are expressed in flower buds, the tissue used for the initial analysis of base-paired RNAs. Eight of these transcription units are expressed in all five tissues, and three are expressed only in unopened flower buds (Figure 6E–6I; Figure S6H, S6I, S6J, S6N, S6O, and S6P). Two of these latter transcripts are also the source of smRNAs (Figure 6A and Figure S6A; Table S5). Overall, our findings reveal a large collection of novel, structured RNAs in *Arabidopsis* flower buds, many of which have evolutionarily conserved functions in land plants (Figure 5B, intergenic).

### Using dsRNA–seq data to produce models of mRNA secondary structure genome-wide

**Figure 7:**
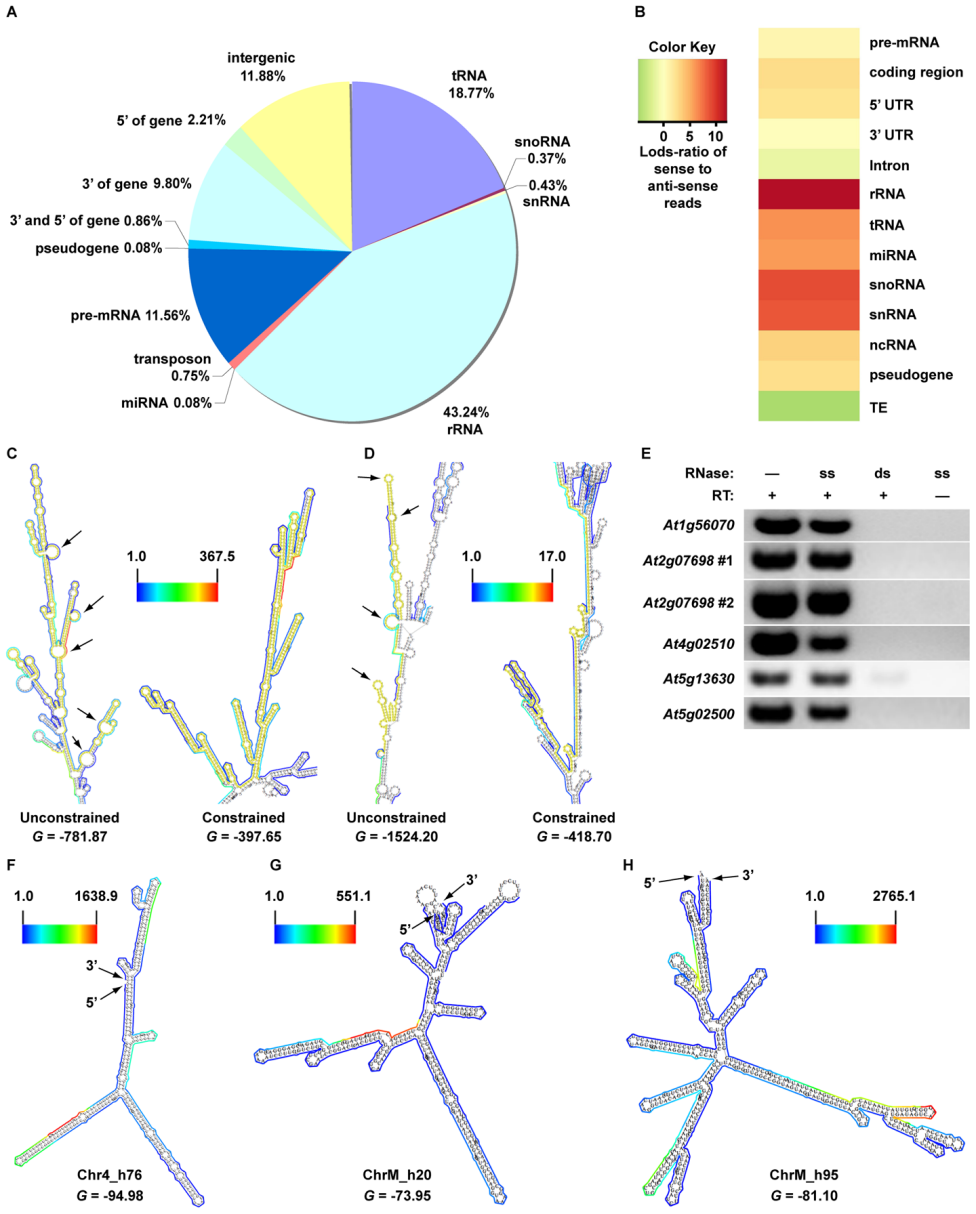
A sequencing-based approach to interrogate mRNA secondary structure genome-wide. (A) Classification of genome-matching dsRNA-seq reads after two rounds of rRNA-depletions (2X Ribominus approach). (B) The heatmap indicates the strand bias of 2X Ribominus dsRNA-seq reads with respect to specific classes of RNA molecules. The color intensities indicate the degree of strand bias as specified by a normalized Lods-ratio value of sense/anti-sense mapping reads (red, sense; green, antisense; yellow, unbiased). TE, transposable element. (C, D) Models of secondary structure for *Arabidopsis* (C) *At2g07698* and (D) *At4g02510* transcripts determined by default (unconstrained) or dsRNA-seq constrained parameters for RNAfold (screenshots from the structural viewer at http://tesla.pcbi.upenn.edu/annoj_at9/). The sequences interrogated in (E) (At2g07698 #1 and At4g02510) are highlighted in yellow. The scale bar between the two models indicates the read counts that are normalized by the length of sequenced bases for the transcript. Black arrows indicate RNA loops that are >5 nt within the yellow shaded portions of the models. *G* denotes the Gibb's free energy value (kilocalories/mole) for the corresponding RNA secondary structure model. (E) Random-primed RT-PCR analysis of dsRNA ‘hotspots’ from *At5g56070*, *At2g07698* (2), *At4g02510*, *At5g13630*, and *At5g02500* after treatment of total RNA samples with either a single-stranded (ss) or double-strand RNase (ds). Samples that were not treated with reverse transcriptase (RT -) or either RNase (-) serve as controls for this experiment. (F–H) Models of secondary structure for *Arabidopsis* (D) chr4_h76 (chr4: nt 1476284–1476589), (E) chrM_h20 (chrM: nt 46875–47251), and (F) chrM_h95 (chrM: nt 334344–334833) novel intergenic transcripts determined by dsRNA-seq constrained parameters for RNAfold (screenshots from the structural viewer at http://tesla.pcbi.upenn.edu/annoj_at9/). The scale bar to the left (F, G) or right (H) of each model indicates the read counts that are normalized by the length of sequenced bases for the transcript. *G* denotes the Gibb's free energy value (kilocalories/mole) for the corresponding RNA secondary structure model.

In principle, dsRNA-seq data should reveal the pairing status of all sequences within expressed mRNA molecules (Figure 1). If this is true, this approach can be used to generate and/or validate secondary structural predictions on a genome-wide scale. To test this hypothesis, we employed a novel methodology that produces structural models using sequence data obtained with a dsRNA-seq approach. For this analysis, we used sequence data obtained from samples that were processed using two rRNA-depletion steps (2X Ribominus approach (see Text S1; Figure S7)). We used this dataset because - although incredibly similar to the normal dsRNA-seq approach (see Text S1) - it is enriched for sense-strand mRNA sequences (Figure 7A and 7B, Figure S4D, and Figure S7), increasing the likelihood of generating useful secondary structure models. This mRNA secondary structure analysis revealed base-pairing differences between the structural models produced by the RNAfold program of the Vienna package (http://www.tbi.univie.ac.at/~ivo/RNA/) with and without dsRNA-seq constraints. Many regions that were predicted not to base-pair, but to form large loops and open regions by non-constrained RNAfold were more highly paired when constrained, and vice versa (see Figure 7C and 7D, http://tesla.pcbi.upenn.edu/annoj_at9/).

To test the ability of our structural modeling approach to predict highly base-paired regions, we characterized significantly paired regions of mRNAs (as determined by our methodology) (Figure 7C and 7D, see yellow regions) by reverse transcription (RT) polymerase chain reaction (RT-PCR) after digestion with a single-stranded or double-stranded RNase. We expected that the selected mRNA regions would be sufficiently intact for RT-PCR amplification after treatment with the single-stranded, but not the double-stranded RNase. As predicted, the regions of mRNA molecules determined to be highly base-paired were amplified following treatment with the ssRNase (Figure 7E). Conversely, we could not amplify these same regions after treatment with the dsRNase, which implies that they were completely degraded by this enzyme. These results demonstrated that dsRNA-seq reliably identifies base-paired portions of mRNAs. We also found that the models of secondary structure produced using dsRNA-seq data as constraints are predicted to be stable (Figure 7C, 7D, and 7F–7H, negative *G* values). In total, these results suggest that the constrained secondary structure models are accurate representations of folded RNAs in solution, providing valuable insight into the pairing status of RNA molecules genome-wide.

Finally, we used our mRNA secondary structure prediction methodology to produce folded models for the novel intergenic transcripts identified by the RNA-seq approach (Figure 6 and Figure S6). These structural models indicated that the new RNAs are highly base-paired, and are folded into a diverse array of stable (negative *G* values) secondary structures (Figure 7F–7H). Further evidence that these models are likely to be correct is provided by the observation that we obtained no dsRNA-reads for regions that are predicted to contain large loops by both dsRNA-seq data, as well as the RNAfold program of the Vienna package (http://www.tbi.univie.ac.at/~ivo/RNA/). We believe that these transcriptome-wide mRNA secondary structure models and corresponding web-based viewer (http://tesla.pcbi.upenn.edu/annoj_at9/) will be useful tools for elucidating the function of RNA folding in regulating gene expression and protein translation.

### Conclusions

We describe in this report novel methodologies that produce a comprehensive genomic view of intra- and intermolecular base-paired RNAs at unprecedented resolution. We take advantage of the data from these approaches, which capture intra-molecular base-pairing interactions, to generate models of mRNA secondary structure in solution on a genome-wide scale (Figure 7). Although our methodology reveals the pairing status of RNA molecules in the absence of cellular proteins, previous studies have demonstrated that structural information obtained for RNAs in solution accurately reflects their structure in ribonucleoprotein complexes [3], [45]. Furthermore, our identification of conserved functional RNA domains using dsRNA-seq strongly suggests that RNA molecules are correctly folded into their secondary structure in solution (Figure 5). Overall, our results suggest we have produced highly informative models of mRNA secondary structure on a genome-wide scale for *Arabidopsis*, which can serve as a model for orthologous RNAs from other eukaryotic organisms.

As a resource for the larger community we have made available all sequencing data sets to NCBI Gene Expression OmniBus (GEO), and we have displayed them in a powerful and easy-to-use genome browser, Anno-J (http://tesla.pcbi.upenn.edu/annoj_at9/). Additionally, we have made the models of mRNA secondary structure freely available to the community through a structure viewer that has been incorporated into the dsRNA-seq Anno-J browser. Overall, the methods we have developed, as well as the highly informative sequencing data sets and models of RNA secondary structure that have resulted from this study will contribute positively to future work aimed at illuminating the numerous functions that RNA secondary structure has in regulating eukaryotic gene expression during developmental processes.

## Materials and Methods

### Text S1 information

Further details on the plant materials, experimental procedures, high-throughput sequencing, processing, mapping, and analysis of Illumina GA sequence reads are provided in Text S1. Primers used in this study are listed in Table S7.

### dsRNA–seq library preparation

Briefly, total RNA is subjected to one (1X Ribominus) or two (2X Ribominus) rounds of rRNA depletion as per manufacturer's instructions (Ribominus, Invitrogen (Carlsbad, CA)). Next, these rRNA-depleted RNA samples are treated with a single-strand specific ribonuclease as per manufacturer's instructions (RNase One, Promega (Madison, WI)). The RNA sample is then used as the substrate for sequencing library construction using the Small RNA Sample Prep v1.5 kit (Illumina, San Diego, CA) as per manufacturer's instructions. For more detailed methodology see Text S1 and Figure S1A.

### High-throughput sequencing

smRNA-seq and dsRNA-seq libraries were sequenced using the Illumina Genetic Analyzer II as per manufacturer's instructions (Illumina Inc., San Diego, CA).

### Sequence read processing and mapping

Sequence information was extracted from the image files with the Illumina (San Diego, CA.) base calling software package (GAPipeline version 1.4). Prior to alignment, sequence reads were reduced to a list of only non-redundant (NR) sequences. NR sequences for which a 3′ adapter sequence was observed were truncated up to the junction with the adapter sequence, while sequences without recognizable 3′ adapters were also retained and processed independently. The dsRNA-seq and smRNA-seq reads were then aligned to the *Arabidopsis* genome (TAIR9 assembly). Finally, NR-sequences with their genomic coordinates were combined to form the final dataset. For more detailed methodology see Text S1.

### Identification of dsRNA “hotspots” in the *Arabidopsis* genome

To identify dsRNA ‘hotspots’ in the *Arabidopsis* genome, we utilized a geometric distribution-based approach. For more detailed methodology see Text S1.

### Gene Ontology (GO) enrichment of dsRNA “hotspot”-containing, protein-coding mRNAs

All protein-coding mRNAs overlapping identified dsRNA ‘hotspots’ were subjected to this analysis. Specifically, the GO enrichment analysis was carried out using the GOEAST web-based “Batch-Genes” tool [46].

### Comparative genomics analysis of *Arabidopsis* dsRNA “hotspots”

The plant seven-way comparative genomics analysis was conducted as previously described. (http://genomewiki.ucsc.edu/index.php/Whole_genome_alignment_howto). For more detailed methodology see Text S1.

### RNA structural models

We generated two computational structures for each annotated transcript. The unconstrained structure was obtained by folding with RNAfold v1.8.4 from the Vienna package with default parameters. The constrained structure was obtained with RNAfold using default parameters, but with structural constraints as additional input defined by reads from the dsRNA-seq approach. Specifically, any position covered by at least one mapped dsRNA read was constrained as paired (‘|’ in the structural constraint input); all other positions were left unconstrained (‘.’ in the structural constraint input).

### Anno-J and RNA structure browser

The Anno-J Genome Browser is a REST-based genome annotation visualization program built using Web 2.0 technology. Licensing information and documentation are available at http://www.annoj.org.

We have developed a structure browser enhancement for Anno-J that enables visualization of the mRNA secondary structure models produced as described above. To do this, each predicted model was rendered as a SVG plot using Vienna (http://www.tbi.univie.ac.at/~ivo/RNA/) RNAplot. Reads and other features of interest such as UTR regions for mRNAs were then added to the SVG file. Read counts were normalized by the length of covered nucleotides (e.g. number of nucleotides covered by one or more reads). Users can visualize the structural model for an annotated transcript by selecting the corresponding genomic interval on Anno-J (RNA structures track) or by entering its accession number.

## Supporting Information

Figure S1 Related to Figure 1. (A) Schematic of dsRNA-seq, a novel high-throughput sequencing methodology for identifying and characterizing the dsRNA component of the eukaryotic transcriptome genome-wide. See Text S1 (Supplemental Materials and Methods) for details on the methodology. (B) The relative dsRNA sequence coverage overall (black line) and for 10 classes of RNA molecules (colored lines as specified in legend) as the library subset size changes for the 1X Ribominus dsRNA-seq methodology. (C) The relative dsRNA sequence coverage overall (black line) and for 10 classes of RNA molecules (colored lines as specified in legend) as the library subset size changes for the 2X Ribominus dsRNA-seq methodology.
(8.11 MB TIF)

Figure S2 Related to Figure 2 and Figure 3. (A) The distribution of wild-type Col-0 compared to *rdr6* mutant 1 kb dsRNA-seq differentially expressed (DE) bins along the length of all *Arabidopsis* chromosomes. Each red dot denotes a specific 1 kb dsRNA-seq DE bin (fold change ≥2 and p<.001). Red dots with positive Lods-ratio values are dsRNA-seq DE bins where Col-0> *rdr6*, while negative values denote Col-0< *rdr6*. The blue dots denote known RDR6 TAS substrates as specified. (B) The distribution of wild-type Col-0 compared to *rdr6* mutant 1 kb dsRNA-seq differentially expressed (DE) bins along the length of all *Arabidopsis* chromosomes that correspond to the indicated classes of transcripts. All identified *TAS* transcripts (7/8) are marked with large purple diamonds and labeled. Each green dot denotes a specific 1 kb dsRNA-seq DE bin (fold change ≥2 and p<.001) that corresponds to a protein-coding mRNA. Each black dot denotes a specific 1 kb dsRNA-seq DE bin (fold change ≥2 and p<.001) that corresponds to tandem repeats. Each red dot denotes a specific 1 kb dsRNA-seq DE bin (fold change ≥2 and p<.001) that corresponds to a Gypsy transposon. Each blue dot denotes a specific 1 kb dsRNA-seq DE bin (fold change ≥2 and p<.001) that corresponds to a MuDR transposon. Each fuchsia dot denotes a specific 1 kb dsRNA-seq DE bin (fold change ≥2 and p<.001) that corresponds to a Helitron transposon. All other 1 kb dsRNA-seq genomic bins are marked in grey. (C) The distribution of 1 kb DE bins along all *Arabidopsis* chromosomes where Col-0> *rdr6* in both dsRNA- and smRNA-seq datasets (fold change ≥2 and p<.001). Values above black line denote Lods-ratio for dsRNA-seq DE bins, and values below black line denote results from smRNA-seq analysis. Blue and green dots highlight known RDR6 substrates, TASs and PPRs, respectively. (D) Identifying RDR6 substrates that produce phased smRNAs. (Top) This figure demonstrates the smRNA-seq reads for wild-type Col-0 (red bars) compared to *rdr6* mutant (green bars) plants for an smRNA-producing RDR6 target region in *At2g27400* (*TAS1a*). (Bottom box) The graph shows phase signals from wild-type Col-0 (red line) compared to *rdr6* mutant (green line) smRNA sequence reads for this region of *TAS1a*. Taken together, these results suggest that our analysis can identify phased smRNA-producing substrates of RDR6 in unopened flower buds of *Arabidopsis*.
(7.37 MB TIF)

Figure S3 Related to Figure 2 and Figure 3. (A) The size distribution of dsRNA-seq reads obtained from unopened flower buds of wild-type Col-0 plants using normal and 2X Ribominus dsRNA-seq approaches. The left graph shows the size distribution of all raw dsRNA-seq reads for wild-type Col-0 plants using the normal (yellow bars) and 2X (green bars) Ribominus approaches. The right graph shows the size distribution of all non-redundant (NR) dsRNA-seq reads for wild-type Col-0 plants using the normal (yellow bars) and 2X (green bars) Ribominus approaches. (B) The size distribution of smRNA-seq reads obtained from unopened flower buds of wild-type Col-0 plants (see Figure S8 for analysis). The left graph shows the size distribution of all raw smRNA-seq reads for wild-type Col-0 plants, while the right graph shows the size distribution of all non-redundant (NR) smRNA-seq reads for wild-type Col-0 plants. (C) The size distribution of dsRNA-seq reads obtained from unopened flower buds of *rdr6-11* mutant plants using the normal Ribominus approach. The left graph shows the size distribution of all raw dsRNA-seq reads for *rdr6-11* mutant plants, while the right graph shows the size distribution of all non-redundant (NR) dsRNA-seq reads for *rdr6-11* mutant plants. (D) The size distribution of smRNA-seq reads obtained from unopened flower buds of *rdr6-11* mutant plants. The left graph shows the size distribution of all raw smRNA-seq reads for *rdr6-11* mutant plants, while the right graph shows the size distribution of all non-redundant (NR) smRNA-seq reads for *rdr6-11* mutant plants.
(7.26 MB TIF)

Figure S4 Related to Figure 4, Figure 5, and Figure 7. (A) The distribution of dsRNA ‘hotspots’ identified using the normal (1X Ribominus) dsRNA-seq dataset along the length of all (as specified) *Arabidopsis* chromosomes. Red dots denote specific ‘hotspots’. (B) The distribution of dsRNA ‘hotspots’ identified using the 2X Ribominus dsRNA-seq dataset along the length of all (as specified) *Arabidopsis* chromosomes. Red dots denote specific ‘hotspots’. (C, D) Strand-bias of *Arabidopsis* dsRNA ‘hotspots’. (C) The heatmap indicates the strand bias of dsRNA ‘hotspots’ identified with the 1X Ribominus dataset with respect to specific classes of RNA molecules. The color intensities indicate the degree of strand bias as specified by a normalized Lods-ratio value of sense/anti-sense mapping reads (red, sense; green, antisense; yellow, unbiased). TE, transposable element. (D) The heatmap indicates the strand bias of dsRNA ‘hotspots’ identified with the 2X Ribominus dataset with respect to specific classes of RNA molecules. The color intensities indicate the degree of strand bias as specified by a normalized Lods-ratio value of sense/anti-sense mapping reads (red, sense; green, antisense; yellow, unbiased). TE, transposable element.
(7.27 MB TIF)

Figure S5 Related to Figure 5 and Figure 7. (A, B) Identification of widespread conserved functionality within non-coding portions (introns) of mRNA. (A) The top figure is a model demonstrating the position of the dsRNA ‘hotspot’ within the 3rd intron (from the 5′ end) of *At1g67430*. The black lines delineate the positions within the intron that are demonstrated in the multiple alignment directly below. The bottom figure is the multiple alignment of the best orthologous sequences from six of the seven interrogated plant species. The black bars below the alignments demonstrate the conservation scores for each nucleotide position within the alignment. The red box delineates the position of the dsRNA ‘hotspot’ identified by our geometric distribution-based analysis. (B) The top figure is a model demonstrating the position of the dsRNA ‘hotspot’ within the 5th intron (from the 5′ end) of *At2g40650*. The black lines delineate the positions within the intron that are demonstrated in the multiple alignment directly below. The bottom figure is the multiple alignment of the best orthologous sequences from all seven interrogated plant species. The black bars below the alignments demonstrate the conservation scores for each nucleotide position within the alignment. The red box delineates the position of the dsRNA ‘hotspot’ identified by our geometric distribution-based analysis. (C, D) Identification of widespread conserved functionality within non-coding portions of mRNA (introns, 3′ and 5′ UTRs), intergenic regions, and transposons. (C, D) The average conservation scores (consScore) calculated using a seven-way comparative genomics analysis of dsRNA ‘hotspots’ (green bars) or their flanking regions (yellow bars) in specific portions (coding (exons), 5′ UTR, 3′ UTR, and introns) of pre-mRNAs (C), as well as intergenic regions and tranposons (TE) (D) from the 2X Ribominus approach.
(7.91 MB TIF)

Figure S6 Related to Figure 6. Identification of novel, highly structured RNAs using dsRNA-seq. (A–D) Four examples of intergenic, highly base-paired transcripts (screenshots from http://tesla.pcbi.upenn.edu/annoj_at9). W (red bars) and C (green bars) indicate signal from Watson and Crick strands, respectively. (A) Two intergenic dsRNA ‘hotspots’ (h348 and h349) found between *At2g06555* and *At2g06560*. (B) A novel, base-paired RNA on Chr. 4 between *At4g03360* and *At4g03370*. (C) A Chr. M intergenic dsRNA ‘hotspot’ between *AtMg00160* and *AtMg00170* (D) An example of a new, highly structured RNA from Chr. M that lies between *AtMg01330* and *AtMg01340*. It is of note that these figures demonstrate a more zoomed in representation of the genomic loci that can be seen in Figure 6. (E–G) Three additional examples of intergenic, highly base-paired transcripts (screenshots from http://tesla.pcbi.upenn.edu/annoj_at9). W (red bars) and C (green bars) indicate signal from Watson and Crick strands, respectively. (E) An intergenic dsRNA ‘hotspot’ found between *At1g66400* and *At1g66410*. (F) A novel, base-paired RNA on Chr. 5 between *At5g51670* and *At5g51680*. (G) A Chr. 5 intergenic dsRNA ‘hotspot’ between *At5g54180* and *At5g54190*. (H–J) Random-primed RT-PCR analysis of the novel, base-paired RNAs that are pictured in E–G using five different *Arabidopsis* tissues (leaf blades, leaf petioles, cauline leaves, stems, and unopened flower bud clusters). (H–J) correspond to (E–G), respectively. Unopened flower bud RNA samples that were not treated with reverse transcriptase serve as controls for this experiment. (K–M) Three additional examples of intergenic, highly base-paired transcripts (screenshots from http://tesla.pcbi.upenn.edu/annoj_at9). W (red bars) and C (green bars) indicate signal from Watson and Crick strands, respectively. (K) An intergenic dsRNA ‘hotspot’ found between *At2g07678* and *At2g07669*. (L) A novel, base-paired RNA on Chr. 2 between *At2g20410* and *At2g20420*. (M) A Chr. 4 intergenic dsRNA ‘hotspot’ between *At4g18422* and *At4g18425*. (N–P) Random-primed RT-PCR analysis of the novel, base-paired RNAs that are pictured in (K–M) using five different *Arabidopsis* tissues (leaf blades, leaf petioles, cauline leaves, stems, and unopened flower bud clusters). (N–P) correspond to (K–M), respectively. Unopened flower bud RNA samples that were not treated with reverse transcriptase serve as controls for this experiment.
(8.61 MB TIF)

Figure S7 Related to Figure 7. Highly base-paired segments of the *Arabidopsis* genome (dsRNA ‘hotspots’). (A) Approximate genomic distribution (∼100 kb resolution) and length of dsRNA ‘hotspots’ along *Arabidopsis* Chr. 1 identified using the 2X Ribominus dataset (B) Classification of dsRNA ‘hotspots’ identified using the 2X Ribominus dataset. TE, transposable element. (C) The 18 most significantly enriched molecular functions for protein-coding mRNAs that contain dsRNA ‘hotspots’ identified using the 2X Ribominus dataset. Red labels indicate nucleic acid biology GO categories. (D) The percent of nucleotides within dsRNA ‘hotspots’ hotspots' identified using the 2X Ribominus dataset that were found to produce smRNAs. The smRNA data used for this analysis is described in Figure S8.
(7.87 MB TIF)

Figure S8 Related to Figure 2, Figure 3, Figure 4, and Figure 6. The smRNA component of the *Arabidopsis* unopened flower bud transcriptome. (A) The pie chart demonstrates the classification of smRNA sequencing data from *Arabidopsis* unopened flower buds. (B) Distribution of smRNA ‘hotspots’ along the length of Chromosome 1. Red dots denote specific smRNA ‘hotspots’. (C) Classification of all smRNA ‘hotspots’ in the *Arabidopsis* unopened flower bud transcriptome. (D) The graph shows the overlap between smRNA ‘hotspots’ and dsRNA-seq data along the length of *Arabidopsis* Chr. 1. Red dots denote smRNA “hotspots” that overlap with dsRNA “hotspots”. Green dots denote smRNA ‘hotspots’ that overlap with dsRNA-seq reads covering non-hotspot genomic regions. (E) The distribution of smRNA ‘hotspots’ along the length of all *Arabidopsis* chromosomes. Red dots denote specific ‘hotspots’.
(7.83 MB TIF)

Figure S9 Related to Figure 4 and Figure S7. (A) The relative highly base-paired RNA (dsRNA ‘hotspot’) coverage overall (black line) and for 10 classes of RNA molecules (colored lines as specified in legend) as the library subset size changes for the 1X Ribominus dsRNA-seq methodology. (B) The relative highly base-paired RNA (dsRNA ‘hotspot’) coverage overall (black line) and for 10 classes of RNA molecules (colored lines as specified in legend) as the library subset size changes for the 2X Ribominus dsRNA-seq methodology. This analysis is not informative for miRNAs because too few or no dsRNA ‘hotspots’ are found in this class of RNA molecules for the normal (1X) or 2X Ribominus approaches, respectively. Therefore, they have been intentionally excluded from these graphs.
(7.81 MB TIF)

Table S1 *Arabidopsis* RDR6 substrates determined using the combination of dsRNA- and smRNA-seq.
(0.06 MB XLS)

Table S2 RDR6 substrates that produce phased siRNAs.
(0.03 MB XLS)

Table S3 Normal (1X Ribominus) dsRNA-seq dsRNA ‘hotspots’.
(2.82 MB XLS)

Table S4 2X Ribominus dsRNA-seq dsRNA ‘hotspots’.
(2.24 MB XLS)

Table S5 Normal (1X Ribominus) dsRNA-seq novel RNAs.
(0.30 MB XLS)

Table S6 2X Ribominus dsRNA-seq novel RNAs.
(0.16 MB XLS)

Table S7 Primers used.
(0.03 MB XLS)

Text S1 Supplemental text.
(1.03 MB DOC)
