# Supplementary figures and images for: Genome-Wide Double-Stranded RNA Sequencing Reveals the Functional Significance of Base-Paired RNAs in Arabidopsis
Source: PLoS Genet. 2010 Sep 30;6(9):e1001141. doi: 10.1371/journal.pgen.1001141 (PMC2947979; doi:10.1371/journal.pgen.1001141)

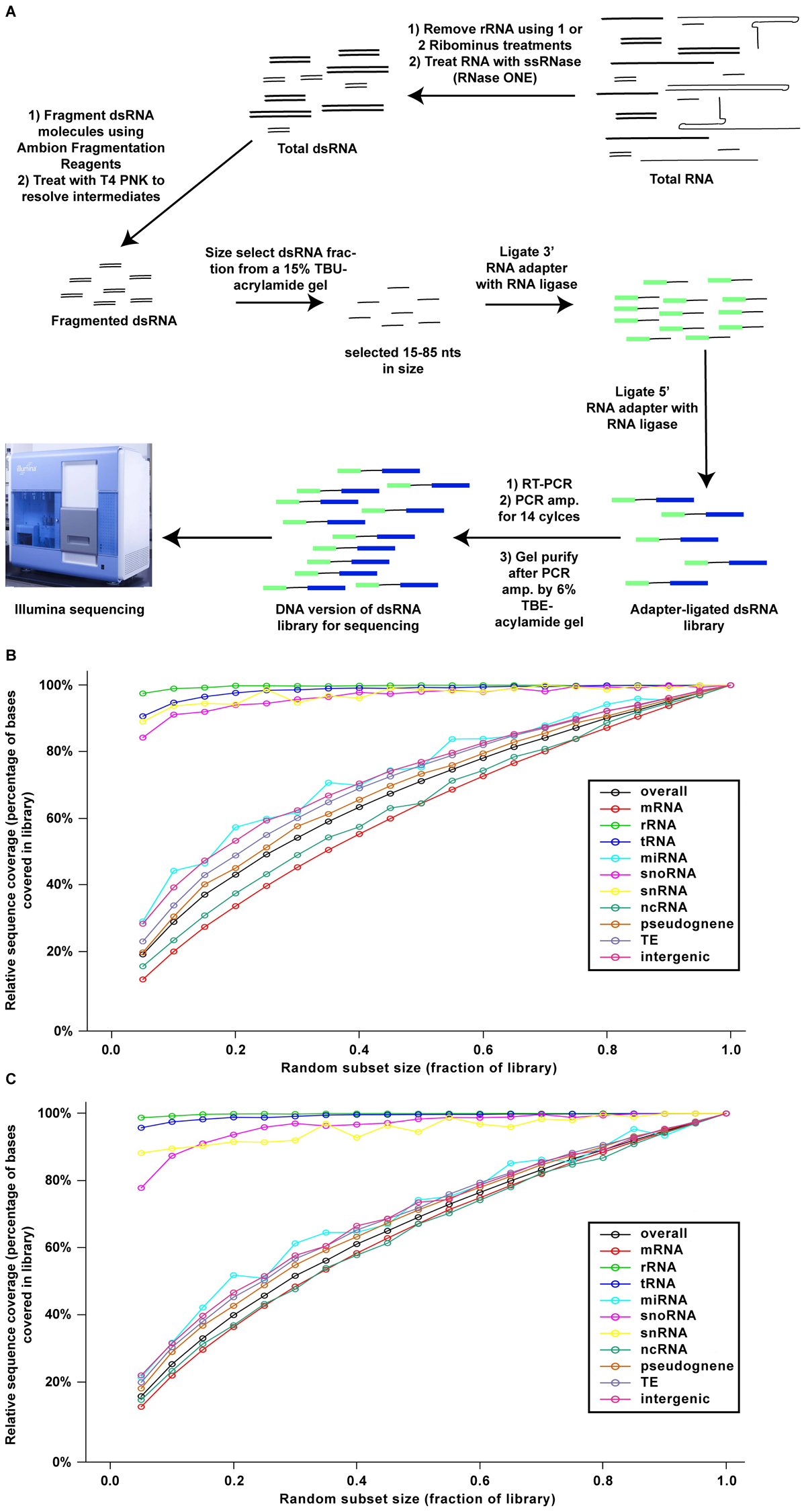

Supplement: Figure S1 — Related to Figure 1. (A) Schematic of dsRNA-seq, a novel high-throughput sequencing methodology for identifying and characterizing the dsRNA component of the eukaryotic transcriptome genome-wide. See Text S1 (Supplemental Materials and Methods) for details on the methodology. (B) The relative dsRNA sequence coverage overall (black line) and for 10 classes of RNA molecules (colored lines as specified in legend) as the library subset size changes for the 1X Ribominus dsRNA-seq methodology. (C) The relative dsRNA sequence coverage overall (black line) and for 10 classes of RNA molecules (colored lines as specified in legend) as the library subset size changes for the 2X Ribominus dsRNA-seq methodology. (8.11 MB TIF) [file pgen.1001141.s001.tif]

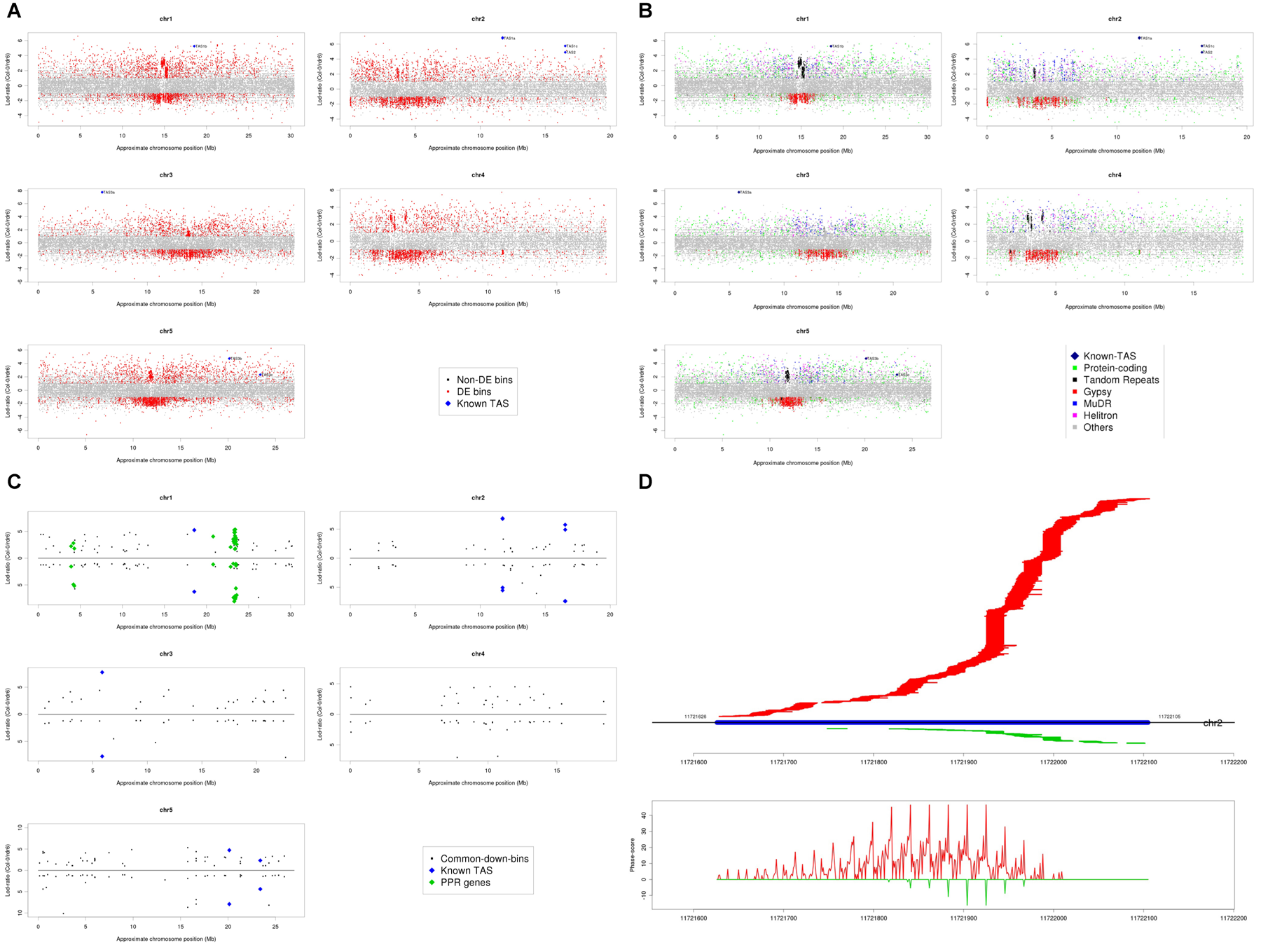

Supplement: Figure S2 — Related to Figure 2 and Figure 3. (A) The distribution of wild-type Col-0 compared to rdr6 mutant 1 kb dsRNA-seq differentially expressed (DE) bins along the length of all Arabidopsis chromosomes. Each red dot denotes a specific 1 kb dsRNA-seq DE bin (fold change ≥2 and p<.001). Red dots with positive Lods-ratio values are dsRNA-seq DE bins where Col-0> rdr6, while negative values denote Col-0< rdr6. The blue dots denote known RDR6 TAS substrates as specified. (B) The distribution of wild-type Col-0 compared to rdr6 mutant 1 kb dsRNA-seq differentially expressed (DE) bins along the length of all Arabidopsis chromosomes that correspond to the indicated classes of transcripts. All identified TAS transcripts (7/8) are marked with large purple diamonds and labeled. Each green dot denotes a specific 1 kb dsRNA-seq DE bin (fold change ≥2 and p<.001) that corresponds to a protein-coding mRNA. Each black dot denotes a specific 1 kb dsRNA-seq DE bin (fold change ≥2 and p<.001) that corresponds to tandem repeats. Each red dot denotes a specific 1 kb dsRNA-seq DE bin (fold change ≥2 and p<.001) that corresponds to a Gypsy transposon. Each blue dot denotes a specific 1 kb dsRNA-seq DE bin (fold change ≥2 and p<.001) that corresponds to a MuDR transposon. Each fuchsia dot denotes a specific 1 kb dsRNA-seq DE bin (fold change ≥2 and p<.001) that corresponds to a Helitron transposon. All other 1 kb dsRNA-seq genomic bins are marked in grey. (C) The distribution of 1 kb DE bins along all Arabidopsis chromosomes where Col-0> rdr6 in both dsRNA- and smRNA-seq datasets (fold change ≥2 and p<.001). Values above black line denote Lods-ratio for dsRNA-seq DE bins, and values below black line denote results from smRNA-seq analysis. Blue and green dots highlight known RDR6 substrates, TASs and PPRs, respectively. (D) Identifying RDR6 substrates that produce phased smRNAs. (Top) This figure demonstrates the smRNA-seq reads for wild-type Col-0 (red bars) compared to rdr6 mutant (green bars) [file pgen.1001141.s002.tif]

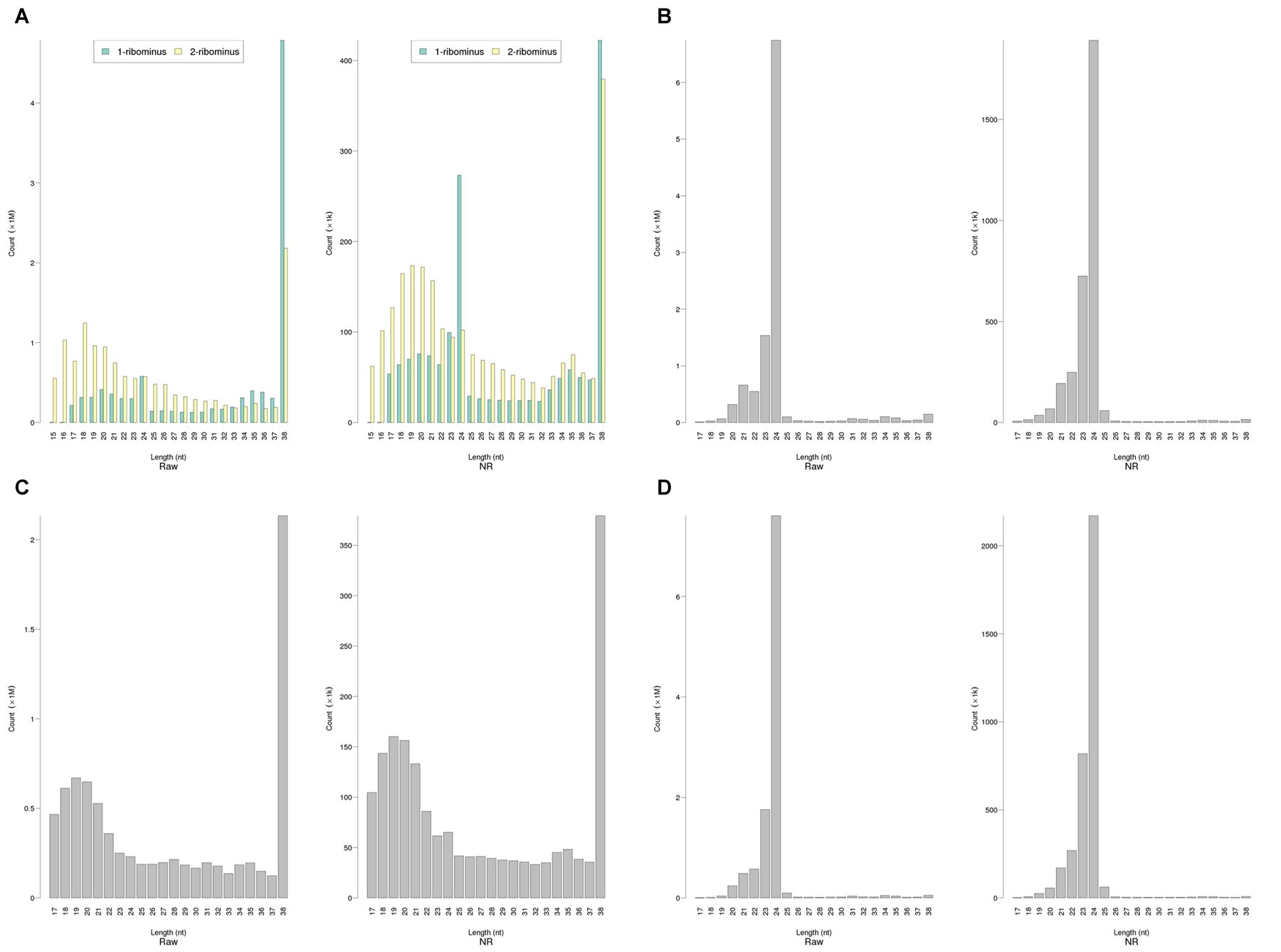

Supplement: Figure S3 — Related to Figure 2 and Figure 3. (A) The size distribution of dsRNA-seq reads obtained from unopened flower buds of wild-type Col-0 plants using normal and 2X Ribominus dsRNA-seq approaches. The left graph shows the size distribution of all raw dsRNA-seq reads for wild-type Col-0 plants using the normal (yellow bars) and 2X (green bars) Ribominus approaches. The right graph shows the size distribution of all non-redundant (NR) dsRNA-seq reads for wild-type Col-0 plants using the normal (yellow bars) and 2X (green bars) Ribominus approaches. (B) The size distribution of smRNA-seq reads obtained from unopened flower buds of wild-type Col-0 plants (see Figure S8 for analysis). The left graph shows the size distribution of all raw smRNA-seq reads for wild-type Col-0 plants, while the right graph shows the size distribution of all non-redundant (NR) smRNA-seq reads for wild-type Col-0 plants. (C) The size distribution of dsRNA-seq reads obtained from unopened flower buds of rdr6-11 mutant plants using the normal Ribominus approach. The left graph shows the size distribution of all raw dsRNA-seq reads for rdr6-11 mutant plants, while the right graph shows the size distribution of all non-redundant (NR) dsRNA-seq reads for rdr6-11 mutant plants. (D) The size distribution of smRNA-seq reads obtained from unopened flower buds of rdr6-11 mutant plants. The left graph shows the size distribution of all raw smRNA-seq reads for rdr6-11 mutant plants, while the right graph shows the size distribution of all non-redundant (NR) smRNA-seq reads for rdr6-11 mutant plants. (7.26 MB TIF) [file pgen.1001141.s003.tif]

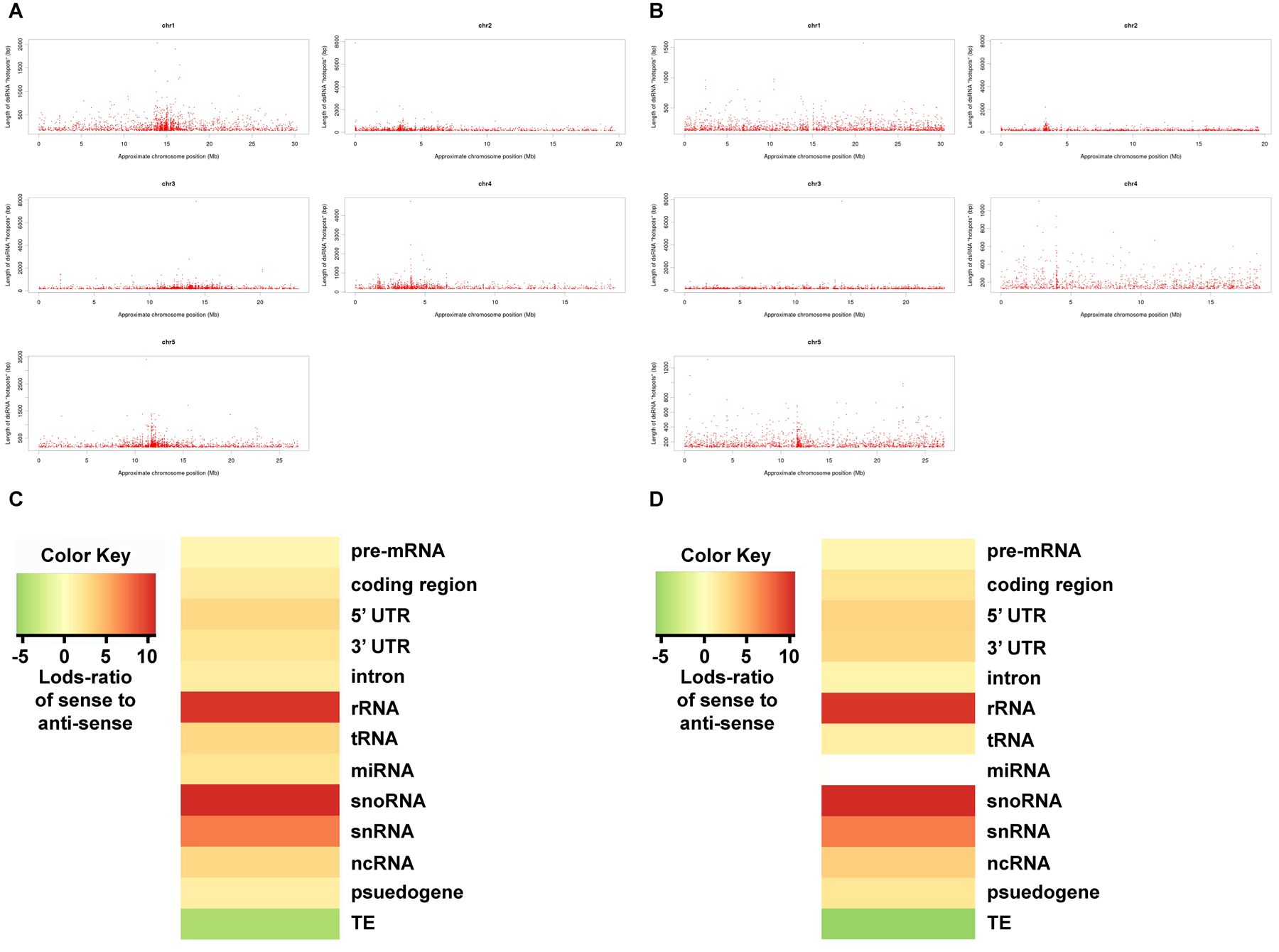

Supplement: Figure S4 — Related to Figure 4, Figure 5, and Figure 7. (A) The distribution of dsRNA ‘hotspots’ identified using the normal (1X Ribominus) dsRNA-seq dataset along the length of all (as specified) Arabidopsis chromosomes. Red dots denote specific ‘hotspots’. (B) The distribution of dsRNA ‘hotspots’ identified using the 2X Ribominus dsRNA-seq dataset along the length of all (as specified) Arabidopsis chromosomes. Red dots denote specific ‘hotspots’. (C, D) Strand-bias of Arabidopsis dsRNA ‘hotspots’. (C) The heatmap indicates the strand bias of dsRNA ‘hotspots’ identified with the 1X Ribominus dataset with respect to specific classes of RNA molecules. The color intensities indicate the degree of strand bias as specified by a normalized Lods-ratio value of sense/anti-sense mapping reads (red, sense; green, antisense; yellow, unbiased). TE, transposable element. (D) The heatmap indicates the strand bias of dsRNA ‘hotspots’ identified with the 2X Ribominus dataset with respect to specific classes of RNA molecules. The color intensities indicate the degree of strand bias as specified by a normalized Lods-ratio value of sense/anti-sense mapping reads (red, sense; green, antisense; yellow, unbiased). TE, transposable element. (7.27 MB TIF) [file pgen.1001141.s004.tif]

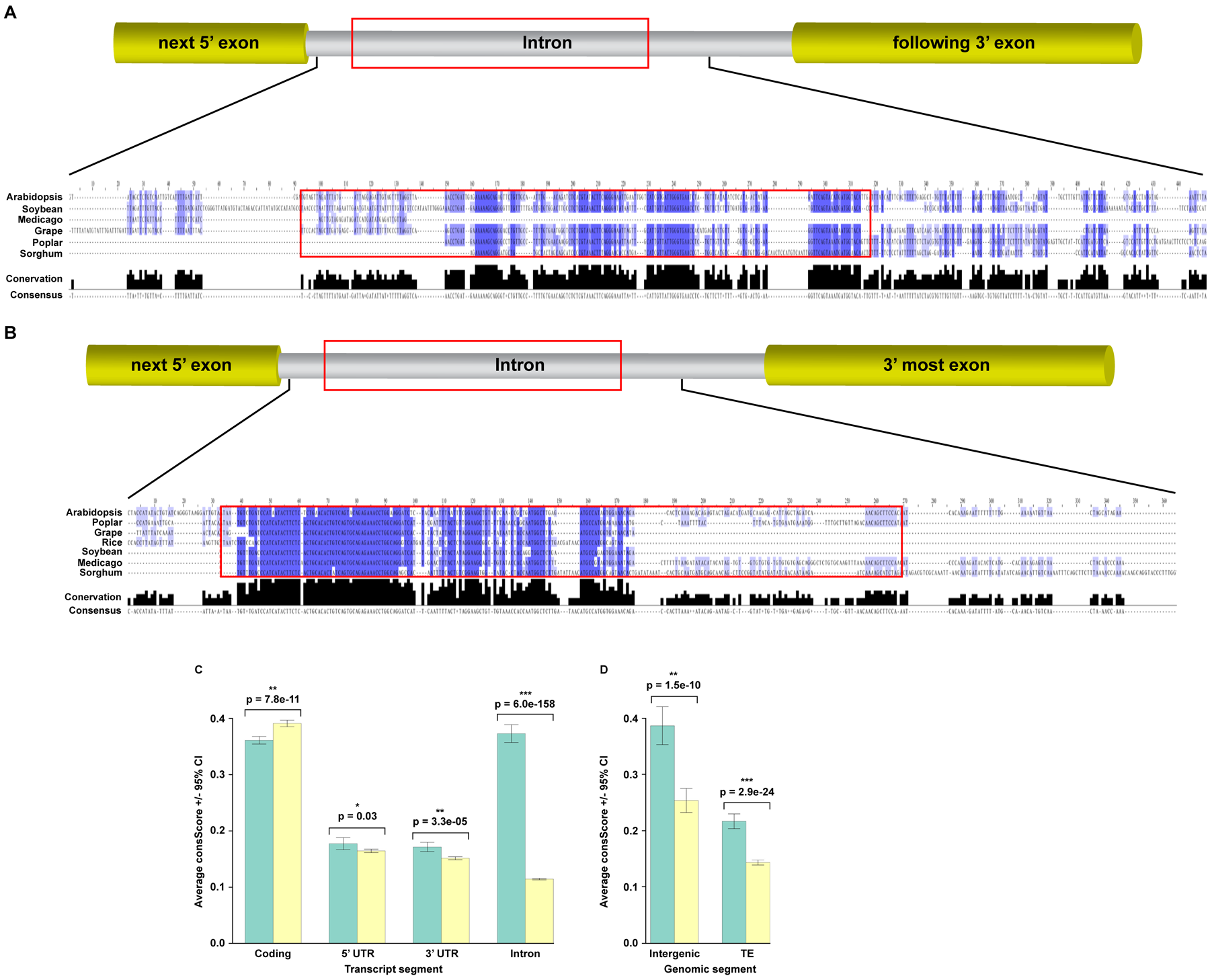

Supplement: Figure S5 — Related to Figure 5 and Figure 7. (A, B) Identification of widespread conserved functionality within non-coding portions (introns) of mRNA. (A) The top figure is a model demonstrating the position of the dsRNA ‘hotspot’ within the 3rd intron (from the 5′ end) of At1g67430. The black lines delineate the positions within the intron that are demonstrated in the multiple alignment directly below. The bottom figure is the multiple alignment of the best orthologous sequences from six of the seven interrogated plant species. The black bars below the alignments demonstrate the conservation scores for each nucleotide position within the alignment. The red box delineates the position of the dsRNA ‘hotspot’ identified by our geometric distribution-based analysis. (B) The top figure is a model demonstrating the position of the dsRNA ‘hotspot’ within the 5th intron (from the 5′ end) of At2g40650. The black lines delineate the positions within the intron that are demonstrated in the multiple alignment directly below. The bottom figure is the multiple alignment of the best orthologous sequences from all seven interrogated plant species. The black bars below the alignments demonstrate the conservation scores for each nucleotide position within the alignment. The red box delineates the position of the dsRNA ‘hotspot’ identified by our geometric distribution-based analysis. (C, D) Identification of widespread conserved functionality within non-coding portions of mRNA (introns, 3′ and 5′ UTRs), intergenic regions, and transposons. (C, D) The average conservation scores (consScore) calculated using a seven-way comparative genomics analysis of dsRNA ‘hotspots’ (green bars) or their flanking regions (yellow bars) in specific portions (coding (exons), 5′ UTR, 3′ UTR, and introns) of pre-mRNAs (C), as well as intergenic regions and tranposons (TE) (D) from the 2X Ribominus approach. (7.91 MB TIF) [file pgen.1001141.s005.tif]

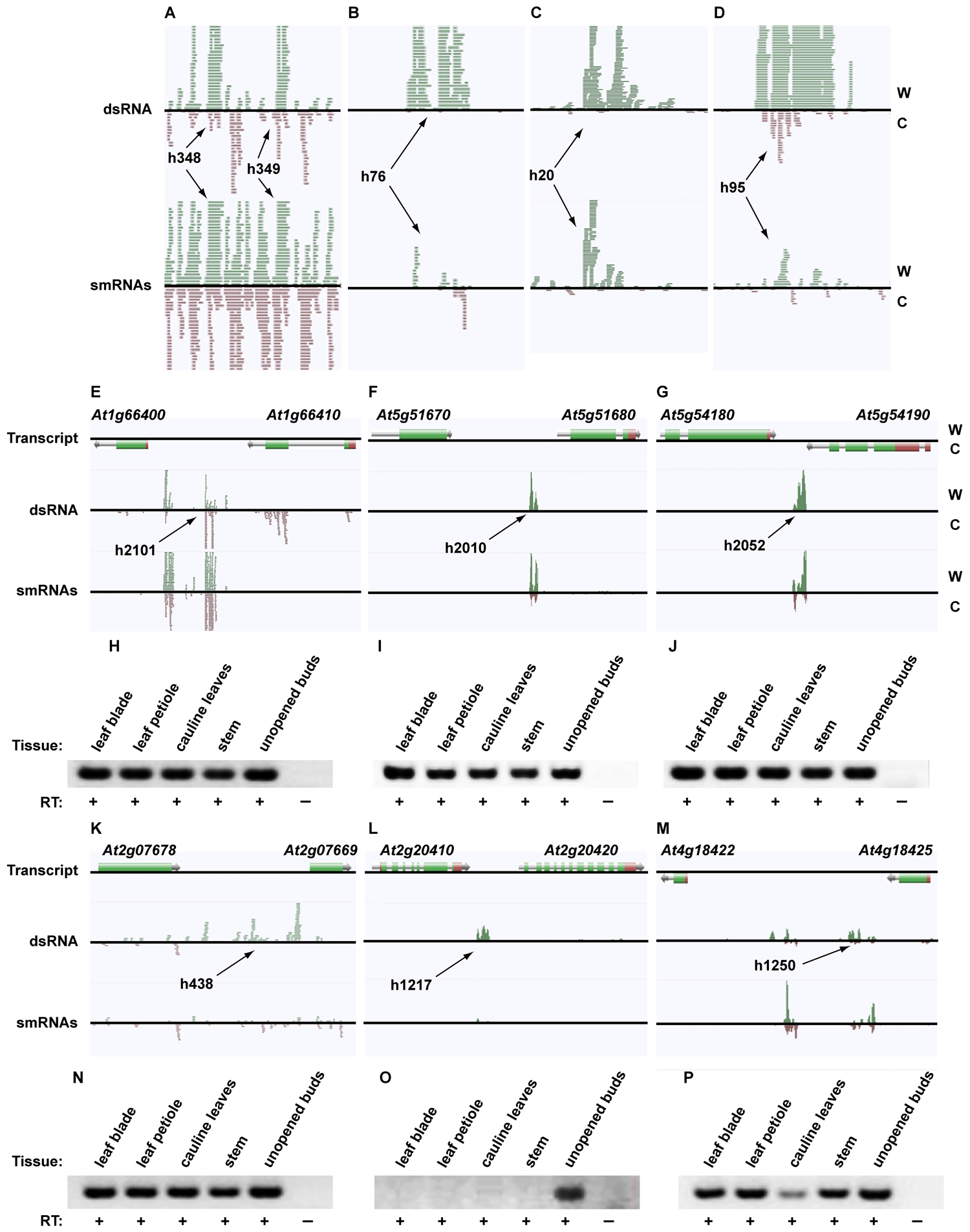

Supplement: Figure S6 — Related to Figure 6. Identification of novel, highly structured RNAs using dsRNA-seq. (A–D) Four examples of intergenic, highly base-paired transcripts (screenshots from http://tesla.pcbi.upenn.edu/annoj_at9). W (red bars) and C (green bars) indicate signal from Watson and Crick strands, respectively. (A) Two intergenic dsRNA ‘hotspots’ (h348 and h349) found between At2g06555 and At2g06560. (B) A novel, base-paired RNA on Chr. 4 between At4g03360 and At4g03370. (C) A Chr. M intergenic dsRNA ‘hotspot’ between AtMg00160 and AtMg00170 (D) An example of a new, highly structured RNA from Chr. M that lies between AtMg01330 and AtMg01340. It is of note that these figures demonstrate a more zoomed in representation of the genomic loci that can be seen in Figure 6. (E–G) Three additional examples of intergenic, highly base-paired transcripts (screenshots from http://tesla.pcbi.upenn.edu/annoj_at9). W (red bars) and C (green bars) indicate signal from Watson and Crick strands, respectively. (E) An intergenic dsRNA ‘hotspot’ found between At1g66400 and At1g66410. (F) A novel, base-paired RNA on Chr. 5 between At5g51670 and At5g51680. (G) A Chr. 5 intergenic dsRNA ‘hotspot’ between At5g54180 and At5g54190. (H–J) Random-primed RT-PCR analysis of the novel, base-paired RNAs that are pictured in E–G using five different Arabidopsis tissues (leaf blades, leaf petioles, cauline leaves, stems, and unopened flower bud clusters). (H–J) correspond to (E–G), respectively. Unopened flower bud RNA samples that were not treated with reverse transcriptase serve as controls for this experiment. (K–M) Three additional examples of intergenic, highly base-paired transcripts (screenshots from http://tesla.pcbi.upenn.edu/annoj_at9). W (red bars) and C (green bars) indicate signal from Watson and Crick strands, respectively. (K) An intergenic dsRNA ‘hotspot’ found between At2g07678 and At2g07669. (L) A novel, base-paired RNA on Chr. 2 between At2g20410 and At2g20420. (M) A Chr. 4 intergenic dsRNA ‘ [file pgen.1001141.s006.tif]

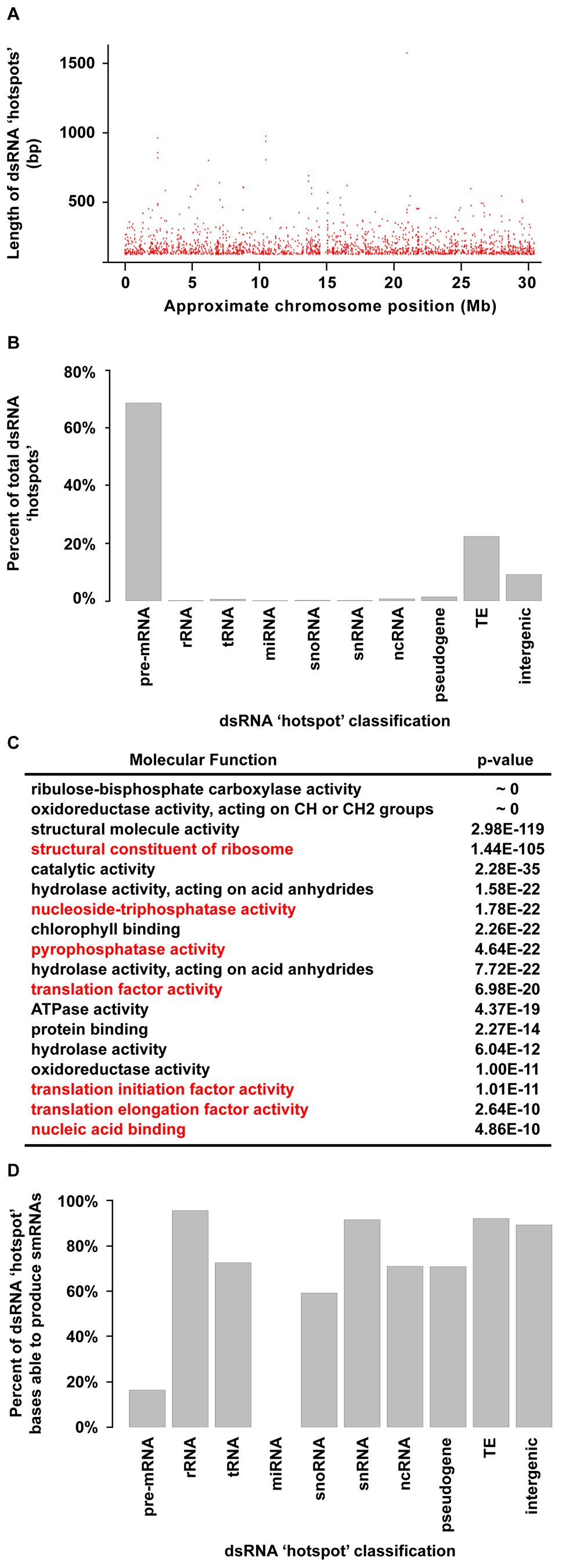

Supplement: Figure S7 — Related to Figure 7. Highly base-paired segments of the Arabidopsis genome (dsRNA ‘hotspots’). (A) Approximate genomic distribution (∼100 kb resolution) and length of dsRNA ‘hotspots’ along Arabidopsis Chr. 1 identified using the 2X Ribominus dataset (B) Classification of dsRNA ‘hotspots’ identified using the 2X Ribominus dataset. TE, transposable element. (C) The 18 most significantly enriched molecular functions for protein-coding mRNAs that contain dsRNA ‘hotspots’ identified using the 2X Ribominus dataset. Red labels indicate nucleic acid biology GO categories. (D) The percent of nucleotides within dsRNA ‘hotspots’ hotspots' identified using the 2X Ribominus dataset that were found to produce smRNAs. The smRNA data used for this analysis is described in Figure S8. (7.87 MB TIF) [file pgen.1001141.s007.tif]

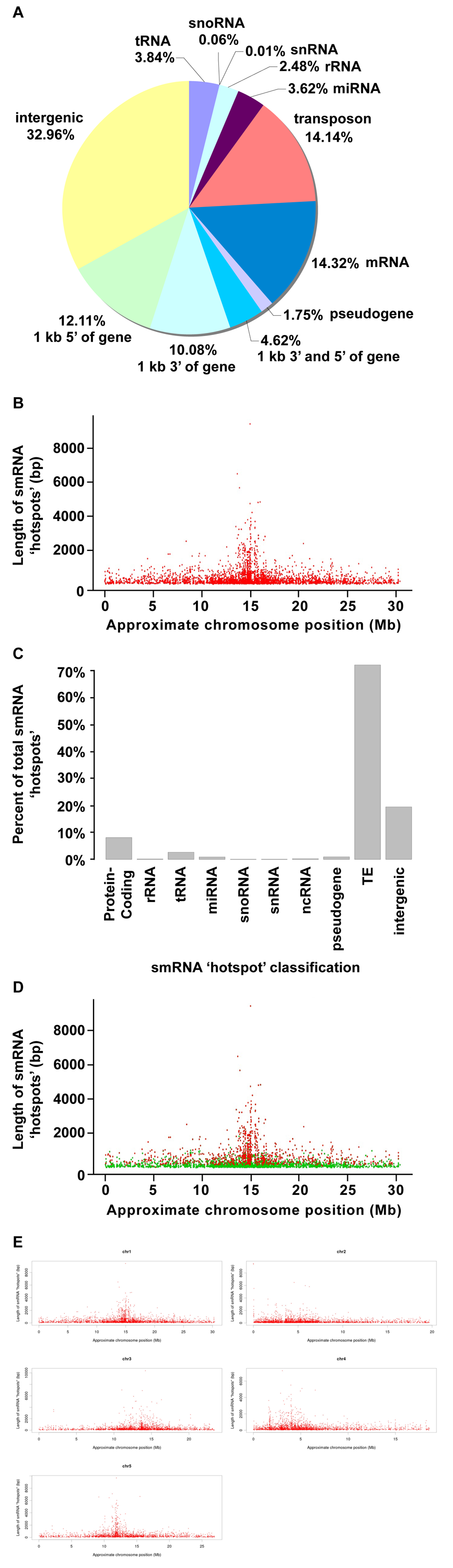

Supplement: Figure S8 — Related to Figure 2, Figure 3, Figure 4, and Figure 6. The smRNA component of the Arabidopsis unopened flower bud transcriptome. (A) The pie chart demonstrates the classification of smRNA sequencing data from Arabidopsis unopened flower buds. (B) Distribution of smRNA ‘hotspots’ along the length of Chromosome 1. Red dots denote specific smRNA ‘hotspots’. (C) Classification of all smRNA ‘hotspots’ in the Arabidopsis unopened flower bud transcriptome. (D) The graph shows the overlap between smRNA ‘hotspots’ and dsRNA-seq data along the length of Arabidopsis Chr. 1. Red dots denote smRNA “hotspots” that overlap with dsRNA “hotspots”. Green dots denote smRNA ‘hotspots’ that overlap with dsRNA-seq reads covering non-hotspot genomic regions. (E) The distribution of smRNA ‘hotspots’ along the length of all Arabidopsis chromosomes. Red dots denote specific ‘hotspots’. (7.83 MB TIF) [file pgen.1001141.s008.tif]

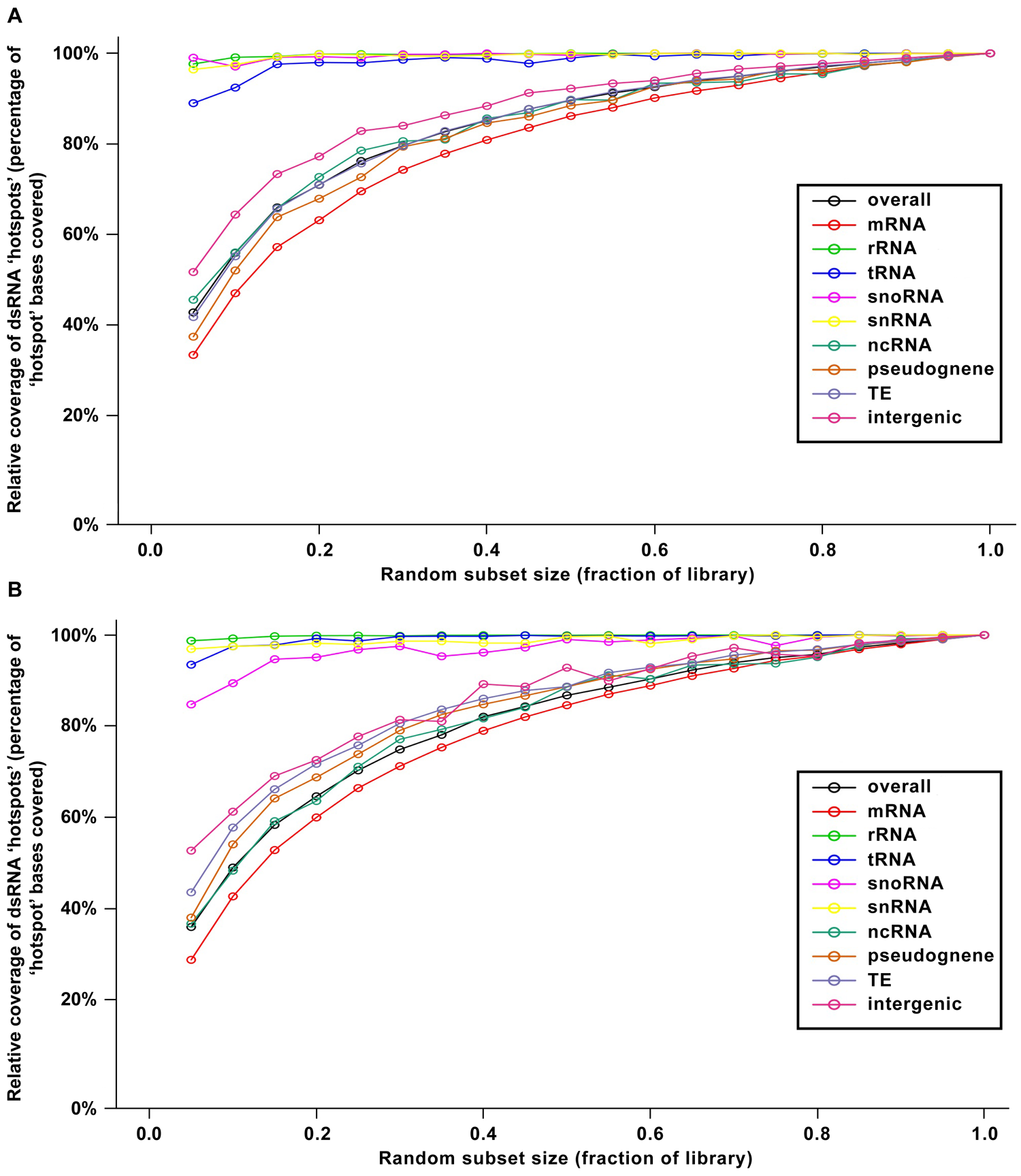

Supplement: Figure S9 — Related to Figure 4 and Figure S7. (A) The relative highly base-paired RNA (dsRNA ‘hotspot’) coverage overall (black line) and for 10 classes of RNA molecules (colored lines as specified in legend) as the library subset size changes for the 1X Ribominus dsRNA-seq methodology. (B) The relative highly base-paired RNA (dsRNA ‘hotspot’) coverage overall (black line) and for 10 classes of RNA molecules (colored lines as specified in legend) as the library subset size changes for the 2X Ribominus dsRNA-seq methodology. This analysis is not informative for miRNAs because too few or no dsRNA ‘hotspots’ are found in this class of RNA molecules for the normal (1X) or 2X Ribominus approaches, respectively. Therefore, they have been intentionally excluded from these graphs. (7.81 MB TIF) [file pgen.1001141.s009.tif]
