# Supplementary material for: Genome-Wide Double-Stranded RNA Sequencing Reveals the Functional Significance of Base-Paired RNAs in Arabidopsis
Source: PLoS Genet. 2010 Sep 30;6(9):e1001141. doi: 10.1371/journal.pgen.1001141 (PMC2947979; doi:10.1371/journal.pgen.1001141)
Supplement: Text S1 — Supplemental text. (1.03 MB DOC) [file pgen.1001141.s017.doc]

**Genome-wide double-stranded RNA sequencing reveals the functional significance of base-paired RNAs in *Arabidopsis***

Qi Zhe^ng1,2^,7, Paul Ryvk^in3^,7, Fan ^L^i3, Isabelle Dragom^i^r1, Otto Valladar^e^s4, Jaime Ya^ng1^,2, Kajia C^a^o4, Li-San Wa^ng2,3,4,5,6^,*, and Brian D. Grego^ry1,2,3,*^

# TEXT S1 INVENTORY

1. Text S1 contains Supplemental Results and is related to Figures 4-7. Supplemental Results describe the results obtained using an additional (2X Ribominus) dsRNA-seq approach. Additionally, there is a Supplemental Results section describing the findings from an analysis performed on a smRNA-seq dataset for wild-type Col-0 that is referred to in Figures 2, 3, 4 and 6.
2. Text S1 contains Supplemental Materials and Methods. Supplemental Materials and Methods describe all methodologies used in for the research in this manuscript thoroughly
3. Text S1 contains Supplemental References.
4. Figure S1 is related to Figure 1 and is a schematic of the dsRNA-seq methodologies employed, developed, and used in this manuscript, as well as an evaluation of total dsRNA coverage by the dsRNA-seq methodology.
5. Figure S2 is related to Figure 2 and contains data complementary to the panels of Figure 2.
6. Figure S3 is related to Figures 2 and 3 and describes the size distribution of the sequencing reads for all dsRNA-seq and smRNA-seq libraries presented in this manuscript
7. Figure S4 is related to Figure 4 and demonstrates the chromosomal distribution and strand bias data for dsRNA ‘hotspots’ identified with both the normal (1X Ribominus) and 2X Ribominus dsRNA-seq approaches.
8. Figure S5 is related to Figure 5 and presents the multiple alignments of two highly conserved intronic dsRNA ‘hotspots’, as well as the results from the comparative genomics analysis of the 2X Ribominus dsRNA-seq dataset.
9. Figure S6 is related to Figure 6 and provides zoomed in screen shots of the novel transcription units shown in Figure 6, as well as characterization of additional novel transcription units.
10. Figure S7 is related to Figure 7 and presents the dsRNA ‘hotspot’ analysis for the 2X Ribominus dsRNA-seq approach.
11. Figure S8 is related to Figures 2, 3, 4 and 6 and presents the analysis of smRNAs from flower buds of wild-type Col-0 that is used to determine the interconnections between dsRNAs and smRNAs in this manuscript.
12. Figure S9 is related to Figures 4 and S7 and presents the analysis of total dsRNA ‘hotspot’ coverage by the normal (1X Ribominus) and 2X Ribominus dsRNA-seq approaches.
13. Table S1 is related to Figures 2 and 3 and contains the pertinent information for the 218 1kilobase (kb) regions that have increased dsRNA and smRNAs in wild-type Col-0 compared to *rdr6* mutant plants.
14. Table S2 is related to Figures 2 and 3 and contains the pertinent information for the 49 1kb regions that produce phased siRNAs, which were identified from a phasing analysis (see above) of the 218 1 kb regions that can be viewed in Table 1.
15. Table S3 is related to Figures 4, 5, and 6 and contains the pertinent information for all dsRNA hotspots identified by the normal (1X Ribominus) dsRNA-seq methodology.
16. Table S4 is related to Figures 7 and S7 and contains the pertinent information for all dsRNA hotspots identified by the 2X Ribominus dsRNA-seq approach that is utilized for RNA secondary structure modeling in this manuscript.
17. Table S5 is related to Figures 4, 5, and 6 and contains the pertinent information for all novel base-paired RNAs identified by the normal (1X Ribominus) dsRNA-seq methodology. This table also contains the overlap information for intergenic dsRNA ‘hotspots’ and smRNAs.
18. Table S6 is related to Figures 7 and S7 and contains the pertinent information for all novel base-paired RNAs identified by the 2X Ribominus dsRNA-seq methodology. This table also contains the overlap information for intergenic dsRNA ‘hotspots’ and smRNAs.
19. Table S7 is related to Figures 3, 6, and 7 and contains the pertinent information for all oligonucleotide primers that were used in this study.

# SUPPLEMENTAL RESULTS

***An additional approach to interrogate the dsRNA component of the Arabidopsis transcriptome***

To obtain a transcriptome-wide view of base-paired RNA (dsRNA) in unopened flower buds of *Arabidopsis thaliana* Col-0 ecotype (hereafter referred to as wild-type Col-0), we married classical nuclease-based structure mapping techniques with high-throughput sequencing technologies (see Figure S1, and Text S1 for details). We also characterized the dsRNA component of the *Arabidopsis* transcriptome after two rounds of ribosomal RNA (rRNA)-depletion, and obtained 31,037,328 raw reads representing 12,243,295 non-redundant (NR) sequences with an average clone-abundance of 2.5. (The size distributions for this dataset can be seen in Figure S3A.) We obtained more sequence reads for the 2X Ribominus approach because we needed increased read coverage of mRNA molecules for our novel mRNA structure modeling approach (Figure 7).

As expected, we found that the majority of our dsRNA sequencing reads for the 2X Ribominus approach corresponded to highly structured classes of RNA molecules that are composed of numerous self-complementary fold-back regions (e.g., rRNA, tRNA, snoRNA, snRNA, etc.), known smRNA-producing loci (e.g., miRNAs), and repetitive elements (e.g., transposons) (Figures 7A and 7B). We also found a large proportion of dsRNAs that correspond to *Arabidopsis* protein-coding transcripts, which likely represent the self-complementary, base-pairing regions that form the secondary structure of mRNA molecules (Figures 7A and 7B). It is of note that dsRNA-seq data mapped to all portions of protein-coding mRNAs, including introns, exons, and both (3’ and 5’) UTRs. Therefore, the dsRNA-seq methodology can identify base-paired regions within both mature and preprocessed mRNA molecules. (For this reason, we refer to protein-coding mRNAs within this manuscript as pre-mRNA.) Overall, our dsRNA-seq approach is highly biased towards classes of RNA molecules that are highly base-paired in nature, which strongly suggests this approach is interrogating the desired component of the transcriptome.

The strand-specific nature of our novel methodology affords us the opportunity to distinguish between intra-molecular fold-back dsRNAs and inter-molecular heteroduplex molecules. To determine the strand bias for the different classes of RNAs captured by dsRNA-seq, we interrogated the ratio of sense versus anti-sense sequencing reads. As indicated by the Log-odds (Lods) values of sense to antisense reads, the majority of RNA classes were strongly enriched for sense-mapping reads, especially for the non-coding RNA classes (rRNA, tRNA, snoRNA, etc.) (Figure 7B). Specifically, functional RNAs (tRNA, miRNA, snoRNA, snRNA, and rRNA) were between 100-1000 fold enriched for the sense compared to the antisense-strand in the 2X Ribominus dsRNA-seq datasets (Figure 7B). Conversely, we identified a strong anti-sense bias in our dsRNA-seq data for transposable element-derived sequences. This may reflect an amplification of the antisense transposon sequence by an RNA-dependent RNA polymerase (RDR) to initiate production of siRNAs and subsequent RNA silencing of these mobile elements. For protein coding regions (exons) of mRNAs, there was a significant sense-strand bias was significantly increased for the 2X Ribominus (~32-fold) compared to the normal (1X) Ribominus (~8-fold) approach. This is likely due to the significant increase in pre-mRNA data obtained utilizing the 2X Ribominus compared to the normal (1X) Ribominus approach (compare Figures 7A to 1A). Note that there is no sense strand bias for dsRNA-seq data that corresponds to introns or 3’UTRs of mRNA molecules (see manuscript for discussion). Taken altogether, these results suggest that our approaches are incredibly similar in their identification of the majority of base-paired RNA molecules (Figures S1B and S1C), which encompass a surprisingly large portion of the *Arabidopsis* genome (~14.4% (17.3 Mb)).

***Identification of dsRNA ‘hotspots’ in the Arabidopsis genome using 2X Ribominus dsRNA-seq***

We next identified regions of the *Arabidopsis* genome that are significantly enriched for base-paired RNA. For this purpose, we used a geometric distribution-based approach to identify unusually long dsRNA molecules (dsRNA ‘hotspots’) based on the average size of dsRNAs computed for each chromosome independently. This analysis revealed 8,965 dsRNA ‘hotspots’ of varying lengths that are scattered along the entire length of all *Arabidopsis* chromosomes for the 2X Ribominus dataset (Figures S7A and S4B; Table S4). Additionally, we found that the highly repetitive, transposon-rich pericentromeric regions of the *Arabidopsis* genome are highly augmented for dsRNA molecules specifically within the normal (1X Ribominus) dsRNA-seq dataset (Figure 4A, 4B, and S4B). Depletion of pericentromeric dsRNA ‘hotspots’ in the 2X Ribominus dataset (Figures S7A, S7B, and S4B) is likely a consequence of the additional heating and renaturation procedures involved in the extra rRNA-depletion, which reduced the representation of intermolecular heteroduplex dsRNA molecules in general (compare Figures 7B and 1B). Such RNA molecules would encompass the heteroduplex dsRNAs synthesized by *Arabidopsis* RDR2 that are necessary for siRNA production and subsequent *cis* transcriptional silencing of transposons and repetitive elements in the pericentromeric regions of *Arabidopsis* chromosomes [1,2,3,4]. Overall, these findings not only substantiate that dsRNA-seq interrogates the desired portion of the transcriptome, but also suggest that, as expected, *Arabidopsis* transposons and repetitive elements are highly enriched in dsRNA on a genome-wide scale.

A classification of *Arabidopsis* dsRNA ‘hotspots’ identified with both the normal (1X Ribominus) and the 2X Ribominus approaches revealed that transposons and protein-coding mRNAs are the two most highly base-paired classes of RNA molecules (Figures 4B and S7B). In fact, we identified 1949 and 3867 protein-coding mRNAs that contained dsRNA ‘hotspots’ for the normal (1X Ribominus) and 2X Ribominus methodologies, respectively (Figures 4C and 4D), and the majority (1476) of these transcripts overlap between the two approaches. Therefore, we determined whether there were any over-represented molecular functions for the encoded proteins of these mRNAs by utilizing classical Gene Ontology (GO) analysis. We found that a number of molecular functions were over-represented in this set of transcripts (Figures 4C and S7C). In particular, we observed that ribulose-bisphosphate carboxylase was the most significantly over-represented molecular function for dsRNA ‘hotspot’-containing mRNAs identified by both the normal (1X Ribominus) and 2X Ribominus methods. Additionally, this analysis revealed that functions involving nucleic acid biology were significantly over-represented by transcripts containing dsRNA ‘hotspots’. These results suggest that transcripts encoding proteins that function in processes involving nucleic acids in eukaryotic cells (e.g., translation, nucleic acid binding, etc.) are highly-enriched in RNA secondary structure through self base-pairing interactions. It is of note that we have obtained similar results for dsRNA ‘hotspot’-containing transcripts of *Drosophila melanogaster* and *Caenhorabitis elegans* (Q.Z. and B.D.G., unpublished data). In total, the GO analysis of dsRNA ‘hotspot’-containing mRNAs suggests a propensity to form complex secondary structure (self base-pairing) is a general feature of eukaryotic transcripts whose encoded proteins function in processes involving nucleic acids. Furthermore, our results hint at a possible feedback regulatory mechanism dependent on the nucleic acid-based function of the encoded protein products and the highly structured nature of the RNA molecule intermediate.

The biogenesis of all functional small silencing RNAs (e.g. miRNAs and siRNAs) requires a dsRNA intermediate. Therefore, we determined the propensity of highly base-paired regions (dsRNA ‘hotspots’) within 10 different classes of RNA molecules to be processed into smRNAs (Figure S7D) using smRNA-seq data for the same RNA samples that were interrogated with the dsRNA-seq methodology (see Figure S8 for smRNA data analysis). We found that the highly base-paired regions within 8 of 10 (there was not data for miRNAs for the 2X Ribominus approach) interrogated RNA categories were extremely likely to be processed into smRNAs, the exception being pre-mRNA molecules (Figure S7D). Although these results were expected for transposable elements and miRNAs, which are known to be smRNA biogenesis substrates, it was surprising that functional RNAs (e.g. rRNA, tRNA, snRNA, etc.) also have a high likelihood of being processed into smRNAs because intramolecular base-pairing interactions are necessary for their function.

***Comparative genomics of 2X Ribominus dsRNA ‘hotspots’ reveals*** *functionality within introns, both UTRs, and intergenic regions of the Arabidopsis genome*

Regulation and maturation of eukaryotic pre-mRNA molecules is intimately linked to proper formation of secondary structure [5,6,7,8]. Such observations suggest that base-paired regions in mRNAs might be functionally conserved throughout evolution. To test this hypothesis, we employed a seven-way comparative genomics approach to evaluate the sequence conservation of dsRNA ‘hotspots’ within exons (coding), introns, and both (5’ and 3’) UTRs of mRNAs compared to all other sequences within the same structural category (‘flanking regions’) for all transcripts. Specifically, an average conservation score (consScore) was calculated for all bases within dsRNA ‘hotspots’ and ‘flanking regions’ for the four structural moieties of every mRNA. The consScores for dsRNA ‘hotspots’ and ‘flanking regions’ were then used to determine whether there is a significant difference in evolutionary conservation. Using this approach, we found that dsRNA ‘hotspots’ in exons are significantly less evolutionarily conserved than ‘flanking regions’ (Figure S5C). These results suggest that intra- and/or intermolecular base-pairing interactions are disfavored in the protein-coding regions of plant mRNAs.

Our comparative genomic analysis of pre-mRNA data obtained with the 2X Ribominus approach revealed that dsRNA ‘hotspots’ are significantly more conserved than ‘flanking regions’ in all three non-protein coding portions of mRNA (see Figures S5C, 5’ UTRs, p = .03; 3’ UTRs, p = 3.5e-5; introns, p = 6.0e-158). Overall, our analyses of the 2X Ribominus dsRNA-seq approach produced strikingly similar results to the normal (1X Ribomimus) methodology. Therefore, these methods can be (for the most part) used interchangeably.

We also noticed that a number of our dsRNA ‘hotspots’ are located in transposons and portions of the genome that do not contain any known elements (intergenic regions). To determine if these highly base-paired RNAs are more evolutionarily conserved, we used our seven-way comparative genomics approach on the 2X Ribominus dsRNA-seq dataset, and found that dsRNA ‘hotspots’ in intergenic regions (p = 1.5e-10) and transposons (p = 2.9e-24) are significantly more conserved than their ‘flanking sequences’ (Figure S5D) just like we observe with the normal (1X Ribominus) dsRNA-seq dataset (Figure 5B). Thus, either dsRNA-seq approach can be utilized to identify novel, highly base-paired, functional RNAs (Figures 5B, S5D, and 6; Tables S5 and S6).

*Identification and characterization of novel, highly base-paired RNAs with conserved functions in land plants using either dsRNA-seq approach*

As stated above, we observed that many dsRNA ‘hotspots’ are located in intergenic regions. Furthermore, these RNAs tend to be under purifying selection, suggesting they are functional (Figures 5B and S5D, intergenic). Specifically, the normal (1X Ribominus) and 2X Ribominus approaches identified a total of 1602 and 814 novel transcription units, respectively (Tables S5 and S6). We found that 656 of these novel RNAs (40.9% and 82.2% from normal and 2X Ribominus, respectively) are identified by both methodologies. Overall, ~50% of the newly identified RNAs are unannotated transposable elements and/or simple repeats (Tables S5 and S6). Thus, we have identified ~700 entirely novel, highly base-paired (structured) RNAs scattered throughout the *Arabidopsis* genome, many of which are functionally conserved in land plants (Figures 5B, S5D, and 6J).

To determine a function for at least a subset of the newly identified RNAs, we interrogated the overlap between these loci and the smRNA component of the *Arabidopsis* unopened flower bud transcriptome (see Figure S8 for smRNA analysis). We found that 1437 (89.7%) and 613 (75.3%) of the novel RNAs identified by the normal (1X Ribominus) and 2X Ribominus approaches, respectively, overlapped with regions of the genome that produce a statistically significant amount of smRNAs (smRNA ‘hotspots’, Figure S8, see Supplemental Materials and Methods for description of analysis) (Figures 6 and S6; Tables S5 and S6). These findings demonstrate that the majority of novel transcription units identified by either dsRNA-seq approach are base-paired precursors of smRNA biogenesis that have not been previously identified or characterized. Furthermore, these RNAs tend to produce smRNAs with evolutionarily conserved functions in land plants. Overall, our sequencing data provides an important informational resource for future studies on smRNA biogenesis and function in land plants. Furthermore, our results suggest that dsRNA-seq is a powerful tool for characterizing the RNA intermediates of smRNA processing, and this information is necessary to increase the utility of RNA silencing as a tool for genetic manipulation of crop plants and human disease (e.g. cancer) therapeutics.

**The smRNA component of the *Arabidopsis* unopened flower bud transcriptome**

We utilized smRNA-seq to characterize the populations of smRNA molecules from unopened flower buds of *Arabidopsis*. Using this approach, we obtained a total of 17,340,638 smRNA reads from our unopened flower bud samples (see the size distribution in Figure S3B). We found that a majority of smRNA sequencing reads mapped to intergenic regions and TEs, whereas only ~ 4% of smRNA sequences matched known miRNAs (Figure S8A). This is expected for the smRNA populations in unopened flower buds of *Arabidopsis*, where it is of the utmost importance that these RNA molecules direct the silencing of repetitive elements and transposons [9,10].

Next, we sought to identify regions of the *Arabidopsis* genome that are significantly enriched in smRNA molecules. To do this, we accounted for the total abundance of sequenced smRNA molecules by applying a modified version of a previously published Poisson distribution-based statistical approach [11]. This analysis revealed that the highly repetitive, TE-rich pericentromeric regions (Figures S8B, S8C, and S8E) are highly augmented for smRNA molecules. Based on the findings of previous studies [9,10], it is not surprising that the majority of smRNA ‘hotspots’ correspond to transposable elements and intergenic regions of the *Arabidopsis* genome (Figures S8B, S8C, and S8E). In fact, our findings are in complete correspondence with our dsRNA ‘hotspot’ analysis for the normal (1X Ribominus) dsRNA-seq approach (Figures 4A and 4B), and provide further support for the hypothesis that pericentromeric dsRNA molecules are truly smRNA-producing substrates that are required for the epigenetic silencing of transposons and repetitive elements littered throughout these genomic regions in *Arabidopsis* [1,2,3,4,12,13,14].

We then investigated whether our dsRNA-seq methodology can identify and characterize the majority of smRNA generating loci genome-wide. By finding the overlap between smRNA “hotspots” and our 1X Ribominus dsRNA-seq dataset, we found that the vast majority (> 90%) of smRNA “hotspots” overlap with either dsRNA “hotspots”, or at the very least dsRNA-seq reads covering non-hotspot genomic regions (Figures S8D). Taken together, these findings demonstrate that our high-throughput dsRNA-seq methodology can be utilized to characterize the dsRNA precursors of most smRNA-producing loci. Overall, our dsRNA-seq data provides an important informational resource for future studies on smRNA biogenesis and function in land plants.

# SUPPLEMENTAL MATERIALS AND METHODS

# Plant materials

The Columbia (Col-0) ecotype of *Arabidopsis* was used in this study. The *rdr6-11* mutant was previously identified [15].

**RNA Analyses**

For all experiments performed herein, immature ﬂower bud clusters, leaves, and all aerial portions of 6-week-old plants were collected for RNA isolation using the miRNeasy Mini Kit (QIAGEN, Valencia, CA). Transcripts were quantiﬁed by RT qPCR using the comparative threshold cycle method (ΔΔC_t_, primers listed in Table S7), using *Actin 2* (*At3g18780*) as the endogenous reference. Random-primed RT PCR was performed using primers listed in Table S7.

dsRNA-seq library preparation

40 μg of total RNA (13.33 μg from each of three biological replicates) is subjected to one (1X Ribominus) or two (2X Ribominus) rounds of rRNA depletion as per manufacturer’s instructions (Ribominus, Invitrogen (Carlsbad, CA)). Next, these rRNA-depleted RNA samples are treated with a single-strand specific ribonuclease as per manufacturer’s instructions (RNase One, Promega (Madison, WI)). dsRNA was then purified using a phenol:chloroform extraction. The purified dsRNA sample is subjected to a fragmentation reaction (Fragmentation Reagents, Applied Biosystems (Foster City, CA)) as per manufacturer’s instructions. To resolve the dsRNAs after single-stranded RNase treatment and fragmentation, they are treated with T4 polynucleotide kinase (T4 PNK, New England Biolabs (Cambridge, MA)) as previously described [16]. The fragmented RNA sample is then used as the substrate for sequencing library construction using the Small RNA Sample Prep v1.5 kit (Illumina, San Diego, CA) as per manufacturer’s instructions. See Figure S1 for a schematic representation of this approach.

**smRNA-seq library preparation**

Briefly, smRNA-seq libraries were produced using the Small RNA Sample Prep v1.5 kit (Illumina, San Diego, CA) as per manufacturers instructions.

**dsRNA and ssRNA RT PCR analysis**

RNase One ssRNase digestion (dsRNA selection) was performed on three 20 μg total RNA samples from wild-type Col-0 unopened flower buds as per manufacturer’s instructions. Following digestion, these three samples were pooled together and purified using a phenol:chloroform extraction. To obtain ssRNA, a dsRNase digestion (RNase V1, (Ambion, Foster City, CA)) was carried on three 20 μg total RNA samples from wild-type Col-0 unopened flower buds as per manufacturer’s instructions. Following digestion, these three samples were pooled together and purified using a phenol:chloroform extraction. Random-primed RT PCR analyses were performed on these digested samples using primers listed in Table S7. This experiment was repeated three times and a representative example can be seen in Figure 7E.

Balanced pre-processing pipeline for mapping dsRNA-seq/smRNA-seq reads

Our dsRNA-seq libraries contain a significant portion of reads in which no 3’-adpater sequences can be found (Figure S3A). To maintain sequence reads that both had discernible 3’-adapter sequences (short reads), as well as reads without 3’-adapters, a balanced pipeline was developed by dividing reads into “trimmed” and “untrimmed” categories according to whether they have detectable 3’-adapter sequences or not, respectively. To begin, all reads are reduced to non-redundant (NR) sequences to minimize the computational requirement for subsequent analysis steps. Then, in order to detect 3’-adapter sequences, all NR-sequences are aligned to the Illumina 3’-adapter version 1.5 sequence using the “cross-match” program from the Phrap/Cross_match/Swat package (<http://www.phrap.org/phredphrapconsed.html>). The alignment parameters for cross-match are carefully tuned to maintain all alignments with less than 6% mis-matches. The cross-match alignment results are parsed using in-house Perl scripts. All NR-sequences that align to ≥ 6 bp of the 3’-adapter sequence at their 3’ end are defined as short reads (with “detectable” 3’-adapter sequence) and are subsequently trimmed at the adapter-sequence boundary. The remaining NR-sequences (without detectable 3’-adapters) remain “untrimmed”.

All trimmed and untrimmed NR-sequences are then aligned to the *Arabidopsis* genome (TAIR9 assembly) using cross-match, again with the parameters to maintain all sequences that align with ≤ 6% mis-matches to the *Arabidopsis* genome. Alignment results for trimmed or untrimmed inputs are then parsed independently using in-house Perl scripts. More specifically, alignments for trimmed sequences are required to extend to the ends of the query sequences, whereas alignments for untrimmed sequences are only required to extend to the imaginary positions of undetectable 3’-adapters (< 6 bp from the 3’ end of the sequence). The true lengths of the untrimmed sequences are also determined in this step by the most-frequent aligned length of all possible alignments to the *Arabidopsis* genome. At last, trimmed and untrimmed NR-sequences as well as their genome loci information are combined to form the final dataset using in-house Perl scripts. The smRNA libraries are pre-processed by this balanced pipeline as well.

Evaluation of coverage by the dsRNA-seq methodology

To evaluate the genome-wide coverage of dsRNA by the dsRNA-seq methodology, we used the Col-0 1X Ribominus dsRNA library as a reference and randomly sampled 19 subsets containing 95% to 5% of total dsRNA-seq reads drawn from the reference dataset in 5% increments. The clone-abundance of the drawn NR-seqs was then re-calculated for each of the random subsets, and the genomic locations of these subsets of NR-seqs were also filtered from the complete set of reference loci. Finally, the base-coverage of NR-seq (unit: bp) for all random subsets, as well as the reference dataset, were calculated for each class of RNA molecules (e.g. rRNA, tRNA, etc.) and for the overall genome. The relative base-coverage of dsRNAs was defined as the fraction of bases covered in each subset compared to the total covered bases of the reference Col-0 1X Ribominus dsRNA dataset. The relative base-coverage of all dsRNA ‘hotspots’ for the two wild-type Col-0 datasets (1X and 2X Ribominus) was achieved by the same method, with the exception that only reads located in dsRNA ‘hotspots’ were used in the analysis.

Estimating the False Discovery Rate (FDR) of dsRNA-seq

The actual FDR of dsRNA-seq is determined by the enzyme efficiency of the ssRNase used (here we used RNaseONE), and is difficult to estimate solely from the sequenced reads. However, we could get an estimate of the upper bound of dsRNA-seq’s FDR by evaluating the proportion of dsRNA reads that have complementary reads in the same library. To begin, we aligned all the genome-mapped reads back to themselves using the NCBI-BLASTN program; the word-size parameter was set to 6 to improve the searching sensitivity of short Illumina reads. By seeking reads with complementary reads other than themselves (align-length ≥ 50% of the read, identity ≥ 85%, mismatches ≤ 10%, and gaps ≤ 5%), we then defined the true dsRNA-reads as those with one or more complementary reads in the same library. Finally, the FDR for the dsRNA-seq methodology was estimated using the Col-0 1X Ribominus dsRNA dataset. It is noteworthy that the FDR value is very likely to be overestimated, since the random fragmentation step in the dsRNA-seq assay can often result in the inability to obtain a complementary read with ≥ 50% overlap even if both reads of the complementary pair are present in the sequencing library. Furthermore, for the lowly expressed dsRNA molecules we could easily sequence only one member of the complementary pair by random chance, while missing the other. Therefore, our estimated FDR for dsRNA-seq likely serves as a conservative upper bound for the technology.

Classification and characterization of dsRNA-seq and smRNA-seq reads

To classify dsRNA-seq and smRNA-seq reads, the GFF annotation file for all *Arabidopsis* genetic elements (protein-coding mRNAs, all non-coding RNAs (rRNAs, tRNAs, miRNAs, pseudogenes, transposable elements, etc.) was downloaded from the TAIR FTP site (TAIR9 release), reformatted using in-house Perl scripts, and loaded in to a local MySQL database. All NR-sequences from dsRNA-seq and smRNA-seq libraries were then classified and annotated according to their location in the *Arabidopsis* genome. For the strand-bias analyses, the sense/antisense relationship for the sequence reads in relation to the strand information for the annotated genetic element at that genomic position is calculated. It is of note that some of our sequencing reads overlap multiple genetic elements, and were correspondingly counted as all pertinent genetic elements for classification purposes.

Classification of intra- and inter- molecular dsRNA duplexes

To assess the proportion of intra- and inter-molecular base-pairing interactions identified by dsRNA-seq, consecutive dsRNA regions/contigs were first grouped into clusters as described in the Identification of dsRNA ‘hotspots’ in the *Arabidopsis* genome section directly below. Then the total mapped dsRNA reads for both the Watson and Crick strand were calculated for each of the dsRNA clusters (duplex) for the wild-type Col-0 1X Ribominus dsRNA-seq library. Finally, dsRNA clusters are defined as intra- or inter-molecular by determining whether they demonstrate comparable amounts of reads from both strands (inter) or not (intra) (Binomial-test, p < 0.05).

Identification of dsRNA ‘hotspots’ in the *Arabidopsis* genome

To identify dsRNA ‘hotspots’ in the *Arabidopsis* genome, dsRNA-seq reads are used to identify contiguous dsRNAs (dsRNA contigs), as well as the remaining ‘ssRNA-regions’ between these base-paired regions. Then, the lengths for dsRNA and ssRNA regions on each chromosome are assumed to both follow a geometric distribution,

$$\begin{matrix} P(X_{i}=k)=(1-p_{i})^{k-1}p_{i} \\ P(X_{i}\leq k)=1-(1-p_{i})^{k} \end{matrix}$$

where $X_{i}$ is the dsRNA/ssRNA region length and $p_{i}$ is the probability for a DNA base to be in dsRNA/ssRNA status for the chromosome ｉ. This assumption holds as long as $p_{i}$ is a constant for every chromosome and the dsRNA/ssRNA status of a DNA base is only dependent on the status of its proceeding base. Using this model, the lengths of dsRNA/ssRNA regions $X_{i}$ are fitted, the parameters $p_{i}$ are estimated, and the confidence intervals of dsRNA/ssRNA region lengths are determined for each chromosome. Finally, dsRNA ‘hotspots’ are identified by selecting “merged” dsRNA contigs with lengths that are longer than statistically expected for that chromosome (see below for method schematic and confidence intervals for all *Arabidopsis* chromosomes used in this analysis). It is of note that “merged” dsRNA regions are combined dsRNA contigs that are separated by ssRNA regions that are shorter than statistically expected as determined by the confidence intervals for ssRNA on that chromosome (see directly below for method schematic and confidence intervals for all *Arabidopsis* chromosomes).


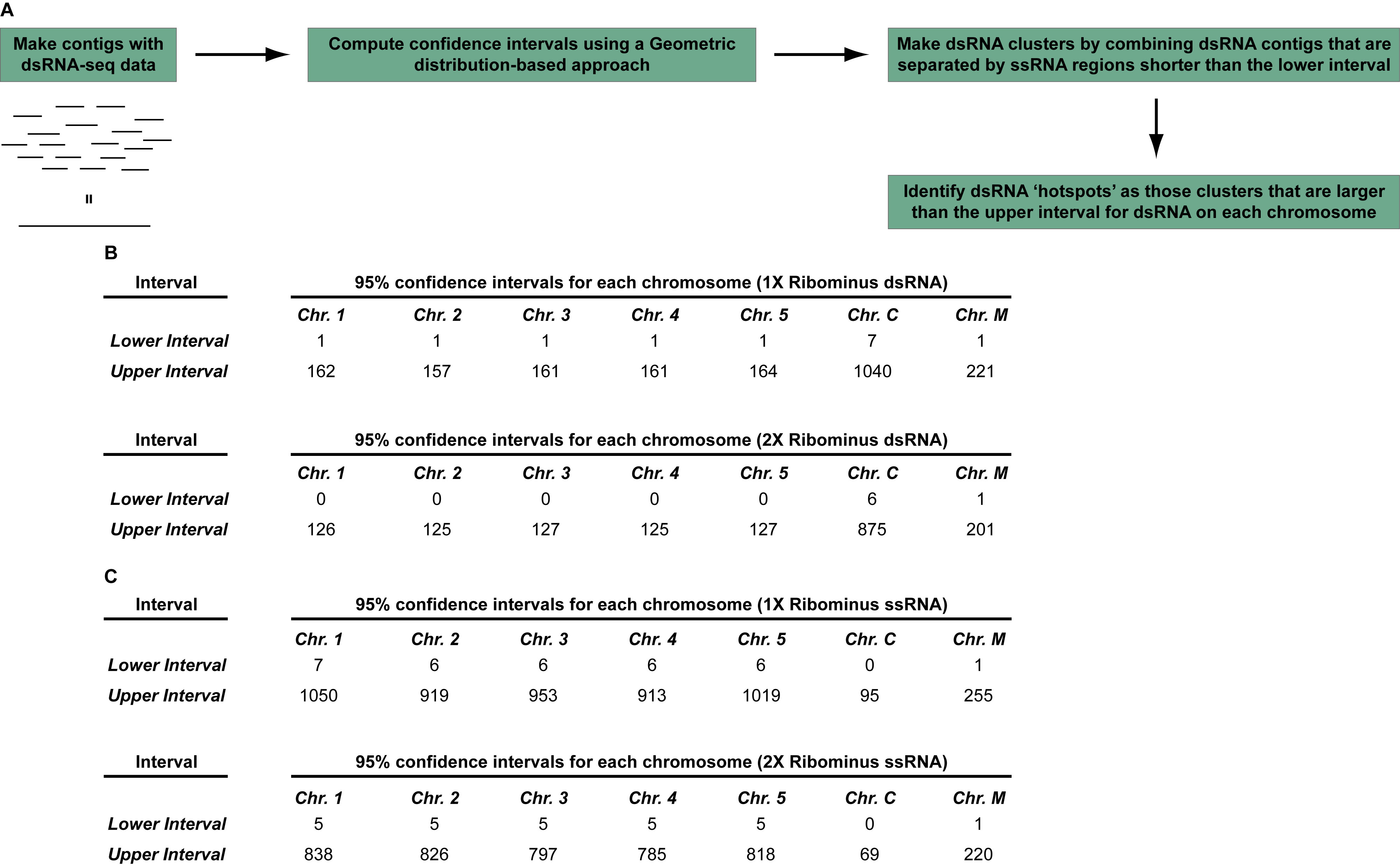


Gene Ontology enrichment analysis for dsRNA ‘hotspots’ harboring protein-coding mRNAs

All protein-coding mRNAs overlapping identified dsRNA ‘hotspots’ were picked out for the analysis. The GO enrichment analysis is carried out using the GOEAST web-based “Batch-Genes” tool [17].

Identification of smRNA ‘hotspots’ in the *Arabidopsis* genome

smRNA ‘hotspots’ were identified differently than the dsRNA ‘hotspots’ in order to take into account expression abundance information of all sequenced smRNA molecules. To begin, consecutive smRNAs are identified on each chromosome and then pre-grouped into smRNA clusters (smRNA contigs). Next, a derived “per-smRNA site” abundance (PSS-abundance) is calculated for all smRNA clusters as $\frac{N_{r}}{L_{c}}\times\bar{X}_{s}$, where $N_{r}$ and $L_{c}$ are the total number of cloned reads and length for this smRNA cluster, respectively, and $\bar{X}_{s}$ stands for the average length of all smRNA reads. Then the derived PSS-abundance on each chromosome is assumed to follow a Poisson distribution:

$$\begin{matrix} P(X_{i}=k)=\frac{\lambda_{^{i}}^{k}}{k!}\cdot e^{-\lambda_{i}} \\ P(X_{i}\leq k)=e^{-\lambda_{i}}\sum_{i=0}^{k} \frac{\lambda_{i}^{k}}{i!}=\frac{\Gamma(\left\lfloor k+1\right\rfloor,\lambda_{i})}{\left\lfloor k\right\rfloor!} \end{matrix}$$

where $X_{i}$ is the derived PSS-abundance and $\lambda_{i}$is the expected number of smRNA reads per smRNA-site on chromosomeｉ. Thus, the derived PSS-abundance data are fitted to this Poisson distribution model, the parameters $\lambda_{i}$ are estimated, and the confidence intervals for PSS-abundance of all smRNA clusters are estimated for each chromosome. Finally, smRNA ‘hotspots’ are identified as smRNA clusters with significantly high PSS-abundance.

Identification of the *Arabidopsis* smRNA-producing RDR6 substrates

dsRNA-seq and smRNA-seq sequencing reads for *rdr6* plants were pre-processed exactly as those for the wild-type Col-0 datasets using our balanced pre-processing pipeline (see above). To find genomic regions that are differentially expressed (DE regions) for both dsRNA and smRNA reads, the *Arabidopsis* genome was cut into 1 kb bins, and the read number of dsRNA or smRNA within each bin was determined. The normalized ratios of cloned read number are calculated for each bin by scaling the read number according to the total read number in Col-0 and *rdr6* libraries. The p-values of significance of the read number between Col-0 and *rdr6* libraries are defined by the posterior probability described previously [18], whose source codes for calculating the posterior probabilities were downloaded and compiled into our in-house C program. The common UP-bins are picked as those that have significantly more sequencing reads in both the dsRNA-seq and smRNA-seq datasets from wild-type Col-0 compared to *rdr6* mutant plants.

Identification of phased-smRNA producing RDR6 substrates

To identify phased-smRNA producing RDR6 substrates, all 218 1 kb regions where dsRNA and smRNAs are more abundant in wild-type Col-0 compared to *rdr6* (Figure 2 and Table S1) were searched for smRNA clusters as defined in the smRNA ‘hotspots’ identification section. Then, a “phase-score”, P, is calculated for every DNA-base within these predetermined smRNA clusters using a modified version of a previously described method [19]: $P=\left\{\begin{matrix} \ln\left[\left(1+\sum_{i=1}^{8} k_{i}\right)^{n-2}\right] (n\geq3) \\ 0 (0\leq n<3) \end{matrix}\right.$, where n and k_i_ have the same meanings as previously described and the phase-circle length L for calculating n and k_i_ is determined by the most frequent length of smRNA reads within this smRNA cluster. At last, phase-scores within a smRNA cluster are tested for hidden periodicity for all smRNA clusters using Fisher’s Exact G Test implemented in the R package “GeneCycle” [20], and phased smRNA-producing RDR6 substrates are selected as smRNA clusters with significantly periodic phase-scores (p < 0.05) within the common UP-bins. It is of note that phase-scores were calculated for both wild-type Col-0 and *rdr6* mutant plants using the same formula, in which the smRNA read number for determining n and k_i_ are normalized first according to the total read numbers of the two smRNA libraries (Figure S2D).

Comparative genomics analysis of dsRNA ‘hotspots in the *Arabidopsis* genome

The overall plant-seven-way comparative genomics analysis was directed by the general instructions listed on the UCSC GenomeBrowser Wiki (<http://genomewiki.ucsc.edu/index.php/Whole_genome_alignment_howto)n> Specifically, all required genome sequences were gathered. The complete genome sequences for the seven plants used in our plant-seven-way comparative genomics analysis were downloaded from various resources, including TAIR (<http://www.arabidopsis.org/>) for *Arabidopsis thaliana* (Ath) genome (TAIR9 assembly); JGI (<http://www.jgi.doe.gov/genome-projects/>) for *Glycine max* (Gma) genome (Glyma1 assembly), *Populus trichocarpa* (Ptr) genome (v 1.0 assembly), and *Sorghum bicolor* (Sbi) genome (v 1.0 assembly); *Medicago* Sequencing Resources (<http://www.medicago.org/genome/>) for *Medicago truncatula* (Mtr) genome (Mt3 assembly); MSU Rice Genome Annotation Project (<http://rice.plantbiology.msu.edu/>) for *Oryza sativa* (Osa) genome (release 6.1), and Genoscope (<http://www.genoscope.cns.fr/spip/>) for *Vitis vinifera* (Vvi) genome (Genoscope 8.4× assembly).

*Masking the genomes*

All seven plant genomes were “soft-masked” for tandem-repeats/simple-repeats using the Tandem-Repeats-Finder program [21]. Besides masking for tandem-repeats, all transposable elements and other simple-repeats were also searched and masked by the RepeatMasker program (http://www.repeatmasker.org/) using repeat libraries from RepBase (release14.06) [22]. All the RepeatMasker masked repetitive elements are loaded into a local MySQL database for usage in later steps.

*Pairwise alignment between the Ath genome and the other plant genomes*

The Ath genome sequence was aligned to the six other plant genomes using the Blastz program [23,24]. This step is computationally intensive, so a Linux cluster was utilized for parallel computation.

*Chaining*

All series of good gapless aligned blocks (chains) from all pairwise alignment results were chained using the UCSC utility programs. All resulting chains are sorted and filtered for netting in the next step.

*Netting*

All the identified sequence chains are constructed into a hierarchical collection of chains (nets), with the highest-scoring non-overlapping chains on top, and their gaps filled in where possible by lower-scoring chains recursively. In addition, transposable elements that emerged after species separation are added back into the nets according to their location identified in the masking step.

*Multiple alignment*

All the nets between the Ath genome and the other six plant genomes were reformatted and progressively multiple-aligned using the Multiz program [25], which was directed by the phylogenic tree of these seven plants as “((((Gma Mtr) Ptr) Ath) Vvi)(Sbi Osa)” in order to generate the final plant multiz-seven-way alignments.

*Building a phylogenic model and estimating consScores*

A phylogenic model was fitted based on the multiple alignments of the seven plant genomes using the phyloFit program in the phastCons package [26] following the phylogenic tree as shown directly above (“((((Gma Mtr) Ptr) Ath) Vvi)(Sbi Osa)”) for which the Generalized time-reversible (GTR or REV) DNA substitution model is utilized. Then the consScores are calculated from the multiz-seven-way alignments using the fitted model for every base involved in the multiz-seven-way alignments. The consScore is in fact a posterior probability for a base to be conserved given the phylogenic model, and thus ranges from 0 to 1.

**SUPPLEMENTAL REFERENCES**

1. Chan SW, Henderson IR, Jacobsen SE (2005) Gardening the genome: DNA methylation in Arabidopsis thaliana. Nat Rev Genet 6: 351-360.

2. Pontier D, Yahubyan G, Vega D, Bulski A, Saez-Vasquez J, et al. (2005) Reinforcement of silencing at transposons and highly repeated sequences requires the concerted action of two distinct RNA polymerases IV in Arabidopsis. Genes Dev 19: 2030-2040.

3. Qi Y, He X, Wang XJ, Kohany O, Jurka J, et al. (2006) Distinct catalytic and non-catalytic roles of ARGONAUTE4 in RNA-directed DNA methylation. Nature 443: 1008-1012.

4. Zheng X, Zhu J, Kapoor A, Zhu JK (2007) Role of Arabidopsis AGO6 in siRNA accumulation, DNA methylation and transcriptional gene silencing. EMBO J 26: 1691-1701.

5. Buratti E, Muro AF, Giombi M, Gherbassi D, Iaconcig A, et al. (2004) RNA folding affects the recruitment of SR proteins by mouse and human polypurinic enhancer elements in the fibronectin EDA exon. Mol Cell Biol 24: 1387-1400.

6. Cooper TA, Wan L, Dreyfuss G (2009) RNA and disease. Cell 136: 777-793.

7. Cruz JA, Westhof E (2009) The dynamic landscapes of RNA architecture. Cell 136: 604-609.

8. Sharp PA (2009) The centrality of RNA. Cell 136: 577-580.

9. Lu C, Tej SS, Luo S, Haudenschild CD, Meyers BC, et al. (2005) Elucidation of the small RNA component of the transcriptome. Science 309: 1567-1569.

10. Lu C, Kulkarni K, Souret FF, MuthuValliappan R, Tej SS, et al. (2006) MicroRNAs and other small RNAs enriched in the Arabidopsis RNA-dependent RNA polymerase-2 mutant. Genome Res 16: 1276-1288.

11. Heisel SE, Zhang Y, Allen E, Guo L, Reynolds TL, et al. (2008) Characterization of unique small RNA populations from rice grain. PLoS One 3: e2871.

12. Herr AJ, Jensen MB, Dalmay T, Baulcombe DC (2005) RNA polymerase IV directs silencing of endogenous DNA. Science 308: 118-120.

13. Kanno T, Huettel B, Mette MF, Aufsatz W, Jaligot E, et al. (2005) Atypical RNA polymerase subunits required for RNA-directed DNA methylation. Nat Genet 37: 761-765.

14. Onodera Y, Haag JR, Ream T, Nunes PC, Pontes O, et al. (2005) Plant nuclear RNA polymerase IV mediates siRNA and DNA methylation-dependent heterochromatin formation. Cell 120: 613-622.

15. Peragine A, Yoshikawa M, Wu G, Albrecht HL, Poethig RS (2004) SGS3 and SGS2/SDE1/RDR6 are required for juvenile development and the production of trans-acting siRNAs in Arabidopsis. Genes Dev 18: 2368-2379.

16. Wang LK, Shuman S (2002) Mutational analysis defines the 5'-kinase and 3'-phosphatase active sites of T4 polynucleotide kinase. Nucleic Acids Res 30: 1073-1080.

17. Zheng Q, Wang XJ (2008) GOEAST: a web-based software toolkit for Gene Ontology enrichment analysis. Nucleic Acids Res 36: W358-363.

18. Audic S, Claverie JM (1997) The significance of digital gene expression profiles. Genome Res 7: 986-995.

19. Howell MD, Fahlgren N, Chapman EJ, Cumbie JS, Sullivan CM, et al. (2007) Genome-wide analysis of the RNA-DEPENDENT RNA POLYMERASE6/DICER-LIKE4 pathway in Arabidopsis reveals dependency on miRNA- and tasiRNA-directed targeting. Plant Cell 19: 926-942.

20. Wichert S, Fokianos K, Strimmer K (2004) Identifying periodically expressed transcripts in microarray time series data. Bioinformatics 20: 5-20.

21. Benson G (1999) Tandem repeats finder: a program to analyze DNA sequences. Nucleic Acids Res 27: 573-580.

22. Jurka J (2000) Repbase update: a database and an electronic journal of repetitive elements. Trends Genet 16: 418-420.

23. Chiaromonte F, Yap VB, Miller W (2002) Scoring pairwise genomic sequence alignments. Pac Symp Biocomput: 115-126.

24. Schwartz S, Kent WJ, Smit A, Zhang Z, Baertsch R, et al. (2003) Human-mouse alignments with BLASTZ. Genome Res 13: 103-107.

25. Blanchette M, Kent WJ, Riemer C, Elnitski L, Smit AF, et al. (2004) Aligning multiple genomic sequences with the threaded blockset aligner. Genome Res 14: 708-715.

26. Siepel A, Bejerano G, Pedersen JS, Hinrichs AS, Hou M, et al. (2005) Evolutionarily conserved elements in vertebrate, insect, worm, and yeast genomes. Genome Res 15: 1034-1050.
